# PBLD inhibits angiogenesis via impeding VEGF/VEGFR2-mediated microenvironmental cross-talk between HCC cells and endothelial cells

**DOI:** 10.1038/s41388-022-02197-x

**Published:** 2022-02-10

**Authors:** Lu Han, Xin Lin, Qun Yan, Chuncai Gu, Mengshu Li, Lei Pan, Yan Meng, Xinmei Zhao, Side Liu, Aimin Li

**Affiliations:** 1 Guangdong Provincial Key Laboratory of Gastroenterology, Department of Gastroenterology, Nanfang Hospital, Southern Medical University, Guangzhou, Guangdong 510515 People’s Republic of China; 2 Department of Gastroenterology, The Second Affiliated Hospital of Guangzhou University of Traditional Chinese Medicine, Guangzhou, Guangdong 510120 People’s Republic of China

**Keywords:** Cancer microenvironment, Liver cancer, Tumour angiogenesis, Non-coding RNAs, Ubiquitylation

## Abstract

Sustained anti-angiogenesis therapy increases the level of tumor hypoxia, leading to increased expression of HIF-1a, thereby contributing to the resistance to anti-angiogenesis therapy in hepatocellular carcinoma (HCC). Here, we report that phenazine biosynthesis-like domain-containing protein (PBLD) inhibits hypoxia-induced angiogenesis via ERK/HIF-1a/VEGF axis in HCC cells. Bioinformatic analysis of the TCGA database and clinical samples validation also identify a negative correlation between PBLD and angiogenesis-related genes expression including HIF-1a. Apart from the downregulation of HIF-1a/VEGF expression in HCC cells, PBLD also blocks VEGF receptor 2 (VEGFR2) on endothelial cells via HCC-derived exosomal miR-940. PBLD also activates TCF4 transcriptional promotion effects on miR-940 by directly interacting with it. Together, PBLD exerts an inhibitory effect on angiogenesis not only via blocking the VEGFR2 expression in endothelial cells, but also through downregulating HIF-1a-induced VEGF expression and secretion in HCC cells. These explorations may provide a theoretical basis for exploring new targets and strategies to overcome resistance to anti-angiogenesis therapy.

## Introduction

Hepatocellular carcinoma (HCC) causes nearly 782,000 deaths worldwide annually and ranks fourth in mortality due to the high rate of recurrence and metastasis of malignant tumors [1]. HCC is a solid tumor with abundant blood supply; therefore, angiogenesis plays an extremely significant role in the occurrence and progression of HCC. Several global multi-center clinical trials have shown that the tyrosine-kinase inhibitors including sorafenib [2, 3], lenvatinib [4], and regorafenib [5] have anti-angiogenic effects, and exert a survival benefit. Sorafenib is the first oral systemic therapy approved in HCC, but it only improves the median survival time by 3~5 months and then develops resistance [6]. The modified tumor microenvironment (TME) after sustained anti-angiogenesis therapy is one of the main reasons accounted for the development of drug resistance [7].

TME is a complicated community that is comprised of cancer cell, microvascular system, extracellular matrix, cancer-associated stromal cells, and their bioactive substances [8]. Pro- and anti-angiogenic factors in TME establish a dynamic equilibrium, which affects angiogenesis via a complex regulatory network [9]. Hypoxia induced by uncontrolled cancer cell growth and sustained anti-angiogenesis therapy greatly increases the secretion of angiogenic factors, which disturbs the equilibrium and shifts it to pro-angiogenesis, thus contributing to the resistance to anti-angiogenesis therapy [10]. Among a multitude of pro-angiogenic factors, the most potent is VEGF, secreted primarily by cancer cells and has the highest specificity for endothelial cells [11]. By binding to its receptors, mainly VEGFR2, on the membrane of endothelial cells, VEGF-VEGFR2 exerts a central regulatory role in the formation of tumor blood vessels [12]. In addition, cancer-derived non-coding RNA and exosomes infiltrated among TME have also been reported to take part in the regulation of angiogenesis.

Exosomes are microvesicles of nanoscale sizes, and transmit various biological molecules between cells, like proteins, mRNA, non-coding RNA, and DNA [13]. Thus, exosomes form a new pattern of intercellular communication and closely participate in various pathologic and physiological processes, including tumor angiogenesis. Exosomes derived from HCC cells transfer vasorin from cancer cells to the vascular endothelial cells, thus, promoting angiogenesis [14]. Another study on HCC also showed that exosomal lncRNA H19 stems from liver cancer cells can remodel TME by promoting angiogenic phenotype [15].

Genomics and proteomics data have revealed the absence of phenazine biosynthesis-like domain-containing protein (PBLD) expression in a variety of tumors, especially HCC [16, 17]. However, there is nearly no in-depth research on molecular mechanisms of PBLD-mediated tumor suppression. In our previous study, we found that PBLD was significantly related to HCC differentiation, clinical stage and patients’ prognosis, and inhibited HCC cells migration and proliferation [18]. In addition, PBLD upregulation induced lower microvessel density (MVD) in a subcutaneously implanted tumor model in nude mice. Further analysis of microarray data also showed the association of PBLD with several tumorigenesis-related signaling pathways including angiogenesis [18]. These findings suggest that tumor suppressor gene PBLD may play a role in tumor angiogenesis.

In this study, we show that overexpression of PBLD in HCC cells impedes the microenvironmental cross-talk between HCC cells and vascular endothelial cells via VEGF-VEGFR2 signaling pathway, thereby leading to the suppression of HCC angiogenesis. Mechanistically, we find that PBLD protects DUSP6 from ubiquitin-proteasome degradation, thus decreasing hypoxia-induced VEGF expression and secretion via the ERK/MAPK-HIF-1a-VEGF axis in HCC cells. We also find that PBLD blocks VEGFR2 expression in endothelial cells via targeting the ETS1/VEGFR2 axis through exosomal miR-940. In general, our findings uncovered the essential role of PBLD in suppressing HCC angiogenesis, thus providing a potential therapeutic target and a new understanding of anti-angiogenesis strategies.

## Results

### Downregulation of PBLD promotes angiogenesis

**Fig. 1:**
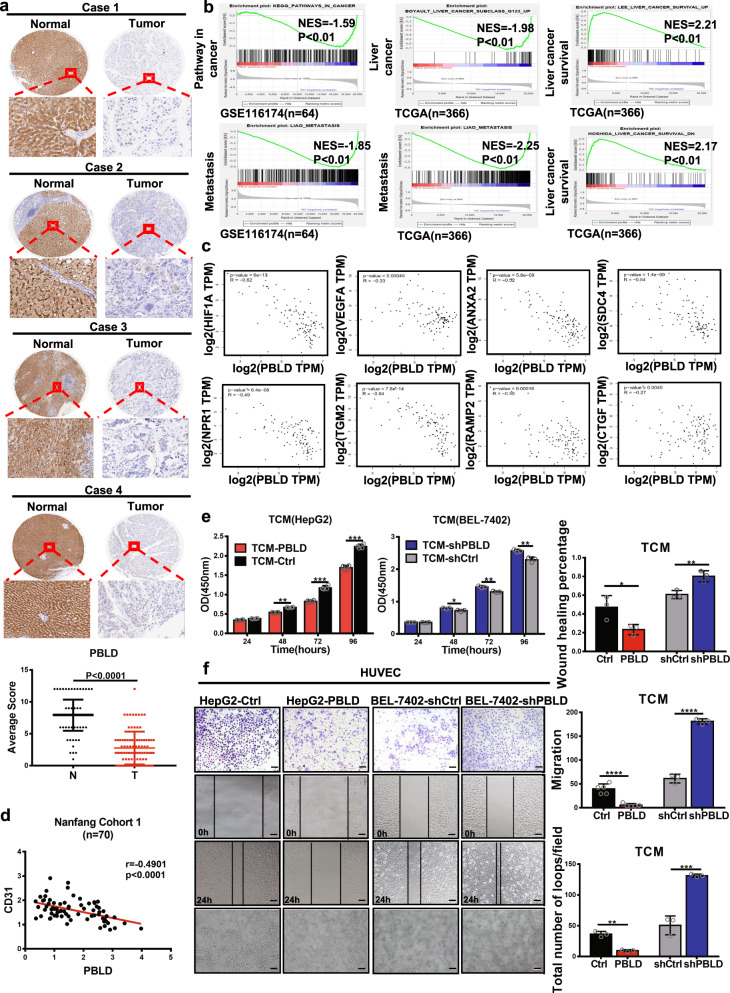
Downregulation of PBLD expression promotes angiogenesis. **a** The representative results of IHC and the expression analysis of PBLD in 90 pairs of HCC tissues and matched normal tissues are shown (*P* < 0.0001, paired *t*-test). **b** GSEA results show that PBLD expression is associated with liver cancer, cancer metastasis and liver cancer survival using TCGA (*n* = 366) and GSE116174 (*n* = 64) datasets. **c** Spearman’s correlation analysis shows negative correlations between PBLD and angiogenesis-related genes using TCGA datasets. **d** Spearman’s correlation analysis showed a negative correlation between PBLD and CD31 mRNA levels in 70 HCC samples from Nanfang cohort 1 (*P* < 0.0001, *r* = –0.4901, *n* = 70). **e** CCK-8 assay revealed that tumor conditional culture medium (TCM) from PBLD-overexpressing cells (HepG2-PBLD) decreased the HUVECs growth, while TCM from PBLD stably silenced HCC cells (BEL-7402-shPBLD) promoted the proliferation in HUVECs. **f** Migration and tube-formation ability detected by Transwell, wound-healing and tube-formation assays in HUVECs treated with TCM from PBLD-upregulated, downregulated and their control HCC cells. Scale bars, 200 µm.

Decreased expression of PBLD in HCC was reconfirmed by immunohistochemistry on a tissue microarray containing 90 pairs of HCC tissues and their matched normal tissues (Fig. 1a and Supplementary Table 1). A gene set enrichment analysis (GSEA) of TCGA and GSE116174 datasets showed that PBLD expression is associated with cancer metastasis and survival (Fig. 1b). Since angiogenesis plays an extremely significant role in the occurrence and metastasis of malignant tumors, further analysis of the TCGA database by GEPIA tool (http://gepia.cancer-pku.cn/) showed the negative correlation of PBLD with angiogenesis-related genes (Fig. 1c). The significantly negative correlation between PBLD and CD31, a surface marker of neovascular endothelial cells, was also validated on clinical samples of our Nanfang cohort (Fig. 1d).

Two HCC cell lines were selected to establish highly expressing (HepG2-PBLD) and silenced cell lines (BEL-7402-shPBLD) of PBLD based on the PBLD constitutive expression as described in our previous study [18]. After culturing for 48 h, the tumor conditional mediums (TCM) from HepG2-PBLD, BEL-7402-shPBLD and their controls (Ctrl) were collected and applied to culture human umbilical vein endothelial cells (HUVECs) for 48 h. Results of CCK-8, wound-healing assay, Transwell assay, and tube-formation assay showed that HepG2-PBLD TCM inhibited HUVECs growth, migration, and tube formation, while opposite effects were observed in BEL-7402-shPBLD TCM (Fig. 1e, f). These results indicate that PBLD inhibits the HCC angiogenesis in vitro, consistent with our previous in vivo experiment [18].

### PBLD inhibits angiogenesis by targeting HIF-1a/VEGF axis

**Fig. 2:**
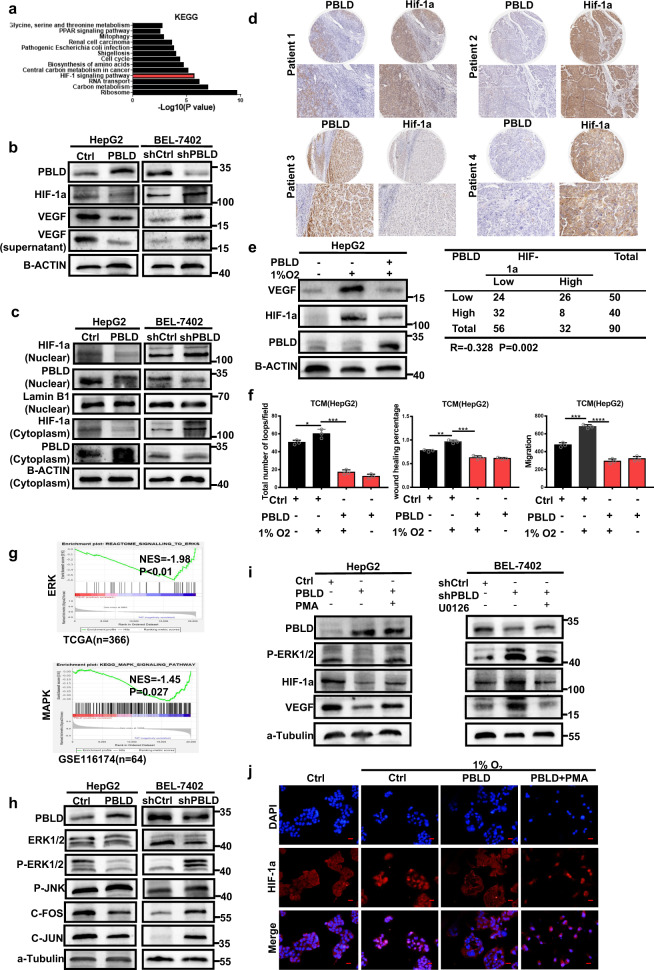
PBLD inhibits angiogenesis by targeting the ERK/HIF-1a/VEGF axis. **a** The KEGG pathway enrichment of significantly altered genes in PBLD-overexpressed HepG2 cells and top 13 pathways are shown. **b** The expression of VEGF and HIF-1a in HepG2 and BEL-7402 cells and the VEGF expression in the culture supernatant were detected by western blot after PBLD upregulation or downregulation. **c** Expression of nuclear or cytoplasmic HIF-1a protein in HepG2 and BEL-7402 cells after PBLD upregulation or downregulation. The control for normalization of nuclear and cytoplasmic separation was LaminB1 and β-actin, respectively. **d** The representative results of IHC and the bivariate correlations analysis of PBLD and HIF-1a in 90 HCC tissues are shown (*r* = –0.328, *P* = 0.002, *n* = 90). **e** The expression of VEGF and HIF-1a was detected by western blot after PBLD upregulation in HepG2 cells under normal or hypoxic (1% O_2_) conditions. **f** Migration and tube-formation abilities detected by Transwell, would-healing and tube-formation assays in HUVECs treated with TCM from PBLD-upregulated and their control HCC cells under normal or hypoxic condition. *P < 0.05, **P < 0.01, ***P < 0.001, and ****P < 0.0001, Student’s *t*-test. **g** The relationship of ERK/MAPK signaling pathway with PBLD expression was shown using TCGA and GSE116174 datasets. **h** Western blot results show the impacts of PBLD on ERK/MAPK signaling pathway. **i** Restoration experiments results show the impacts of ERK dephosphorylation on PBLD-mediated inhibition of HIF-1a and VEGF expression, using PMA (100 nM) and U0126 (10 µM). **j** Representative immunofluorescence staining of HepG2-PBLD, HepG2-Ctrl and HepG2-PBLD+PMA cells stained by HIF-1a antibody under normal or hypoxic conditions. Scale bars, 50 µm.

To delineate mechanisms behind the above-mentioned effects, we performed the Kyoto Encyclopedia of Genes and Genomes pathways enrichment analysis of our previous microarray data (GSE58930). Results showed a significant association between HIF-1a signaling pathways and PBLD expression (Fig. 2a). Subsequent western blot assay confirmed that PBLD overexpression could also decrease the protein levels of HIF-1a and VEGF in HepG2 and BEL-7402 cells (Fig. 2b and Supplementary Fig. 2a). PBLD-mediated reduced VEGF secretion and HIF-1a nuclear translocation were also observed in HCC cells (Fig. 2b, c and Supplementary Fig. 2a, b). The significantly negative correlation between PBLD and HIF-1a expression was also verified by immunohistochemistry on tissue microarray containing 90 HCC tissues (Fig. 2d, Supplementary Fig. 1a, Supplementary Tables 1 and 2). Furthermore, we detect the effects of PBLD on HIF-1a/VEGF expression under hypoxia. Western blot analysis showed that PBLD inhibited the expression of HIF-1a and VEGF induced by hypoxia (Fig. 2e and Supplementary Fig. 2c). Next, HUVECs were cultured in TCM from HepG2 cells treated under hypoxic conditions for 48 h. PBLD overexpression could inhibit hypoxia-induced migration and tube-formation promotion of HUVECs (Fig. 2f and Supplementary Fig. 1b, c). According to these results, PBLD might exert its inhibitory effects on angiogenesis by disrupting the HIF-1a/VEGF pathway.

### PBLD suppresses HIF1-a/VEGF axis via promoting ERK dephosphorylation in HCC

GSEA analysis of TCGA and GSE116174 datasets also suggested a significant correlation between the ERK/MAPK signaling pathway and PBLD expression (Fig. 2g). The negative regulation of ERK/MAPK signaling pathway by PBLD in HCC cells was further verified by western blot (Fig. 2h and Supplementary Fig. 2d). We inhibited ERK1/2 phosphorylation in PBLD-downregulated HCC cell using U0126, a highly selective inhibitor of both MEK1 and MEK2, and found that enhanced expression of HIF-1a and VEGF induced by PBLD downregulation could be inhibited by U0126. Similarly, phorbol 12-myristate 13-acetate (PMA)-mediated activation of ERK phosphorylation could promote the expression of HIF-1a and VEGF suppressed by PBLD overexpression (Fig. 2i and Supplementary Fig. 2e). Furthermore, through immunofluorescence analysis, we observed that PBLD inhibited the nuclear translocation of HIF-1a under hypoxic conditions. However, this suppression effect could be reversed via rescuing the ERK phosphorylation by PMA (Fig. 2j). These results indicate that ERK phosphorylation played a critical role in PBLD-mediated suppression on HIF-1a expression.

### PBLD decreases ERK1/2 phosphorylation by protecting DUSP6 from ubiquitin-proteasome degradation

**Fig. 3:**
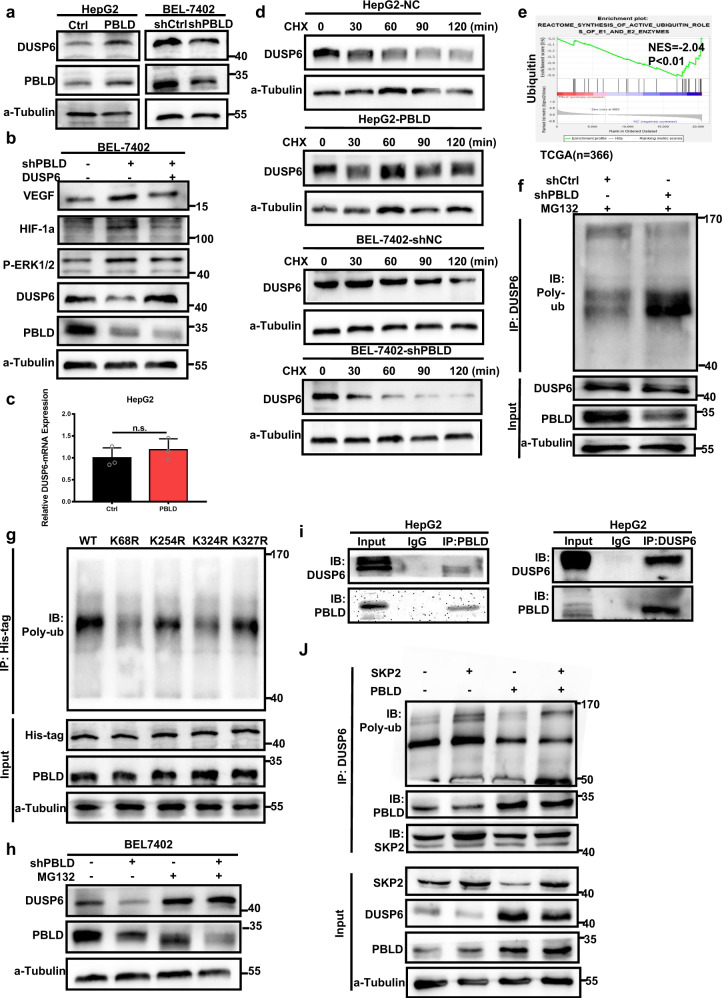
PBLD decreases ERK phosphorylation by maintaining DUSP6 expression. **a** Effects of PBLD on protein expression of DUSP6 in HepG2 and BEL-7402 cells. **b** Western blot analysis of P-ERK, HIF-1a, VEGF, DUSP6, and PBLD in PBLD-downregulated and control group of BEL-7402 cells after transfection with DUSP6 plasmids. **c** The mRNA expression of DUSP6 detected by qRT-PCR in PBLD-upregulated and their control HCC cells. **d** Western blot analyses of DUSP6 levels in PBLD-upregulated and PBLD-downregulated cells, which were treated with 20 µg/mL CHX for the indicated periods of time. **e** GSEA analysis shows a relationship of ubiquitination pathway with PBLD expression using TCGA datasets. **f** Cell lysates of PBLD-downregulated HCC cells were immunoprecipitated with an antibody against DUSP6 and detected by immunoblotting using ubiquitin (Ub)-specific antibody. **g** PBLD-downregulated HCC cells were transfected with vectors expressing the His-tagged DUSP6-WT or DUSP6-mutants (K68R, K254R, K324R, and K327R). Co-immunoprecipitation assays were performed using anti-His antibodies, and detected by immunoblotting with a ubiquitin (Ub)-specific antibody. **h** DUSP6 expression in PBLD-downregulated and control groups of BEL-7402 cells treated with MG132 (10 µM). **i** Co-immunoprecipitation assay to identify the interaction of PBLD with DUSP6 in HepG2 cells. **j** Co-immunoprecipitation assay using DUSP6 antibody to show effects of PBLD on the combination between SKP2 and DUSP6. Western blot analysis of SKP2, DUSP6, and PBLD in PBLD-upregulated and control group of HepG2 cells after transfection with SKP2 plasmids.

The depletion of DUSP6 proteins, which had specificity for dephosphorylation of the ERK family, was observed in BEL-7402-shPBLD, in contrast to that observed in HepG2-PBLD (Fig. 3a and Supplementary Fig. 3a). Furthermore, enhanced HIF-1a/VEGF protein expression and ERK phosphorylation level in BEL-7402-shPBLD were decreased after the restoration of the DUSP6 (Fig. 3b and Supplementary Fig. 3b). Since the mRNA level of DUSP6 was not affected (Fig. 3c), PBLD possibly suppresses the ERK-HIF-1a-VEGF axis through regulation of DUSP6 protein level.

Therefore, DUSP6 protein half-life was determined to investigate whether PBLD maintained DUSP6 expression through modulating its protein stability. After being treated with the protein synthesis inhibitor cycloheximide (CHX), western blot results revealed a shorter DUSP6 half-life in PBLD-downregulated HCC cells than the control cells, while upregulation of PBLD maintained DUSP6 expression (Fig. 3d and Supplementary Fig. 3c). Moreover, GSEA analysis of TCGA datasets indicated a significant correlation between the ubiquitination pathway and PBLD expression (Fig. 3e), and PBLD-knockdown significantly increased the ubiquitination of DUSP6 (Fig. 3f and Supplementary Fig. 3d) in HCC cells. We then retrieved the experimentally identified ubiquitination sites of DUSP6 from the CPLM databases, including four ubiquitinated lysine (K) residues (K68, K254, K324 and K329). As shown in Fig. 3g, K68R and K324R ubiquitin mutations significantly impaired the ubiquitination of DUSP6 compared with that of the wild-type (WT) in PBLD-downregulated cells. We also found rescued DUSP6 protein expression by proteasome inhibitor MG132 (10 µM) in HCC cells (Fig. 3h and Supplementary Fig. 3f). These results indicate that PBLD uses a ubiquitin-proteasome degradation-dependent mechanism in the control of DUSP6. Further co-immunoprecipitation assay revealed that PBLD interacts directly with DUSP6 to maintain its stability (Fig. 3i).

Then, we used Ubibrowser (http://ubibrowser.ncpsb.org/ubibrowser/) to predict the specific E3 ubiquitin ligase for DUSP6 that would mediate its ubiquitination process. Based on the confidence scores, we selected SKP2 with the highest score (Supplementary Table 3). Subsequent western blot assay verified that enhanced expression of SKP2 changed the ubiquitination level of DUSP6 and induced its degradation, while PBLD reversed the effects of SKP2 on DUSP6 (Fig. 3j and Supplementary Fig. 3g). Interestingly, co-immunoprecipitation assay revealed that DUSP6 protein interacts with both SKP2 and PBLD but binds preferentially to PBLD (Fig. 3j and Supplementary Fig. 3g). Hence, PBLD could maintain DUSP6 expression by competitive binding and inhibiting SKP2-induced DUSP6 degradation.

### PBLD-mediated regulation of angiogenesis through exosomes

**Fig. 4:**
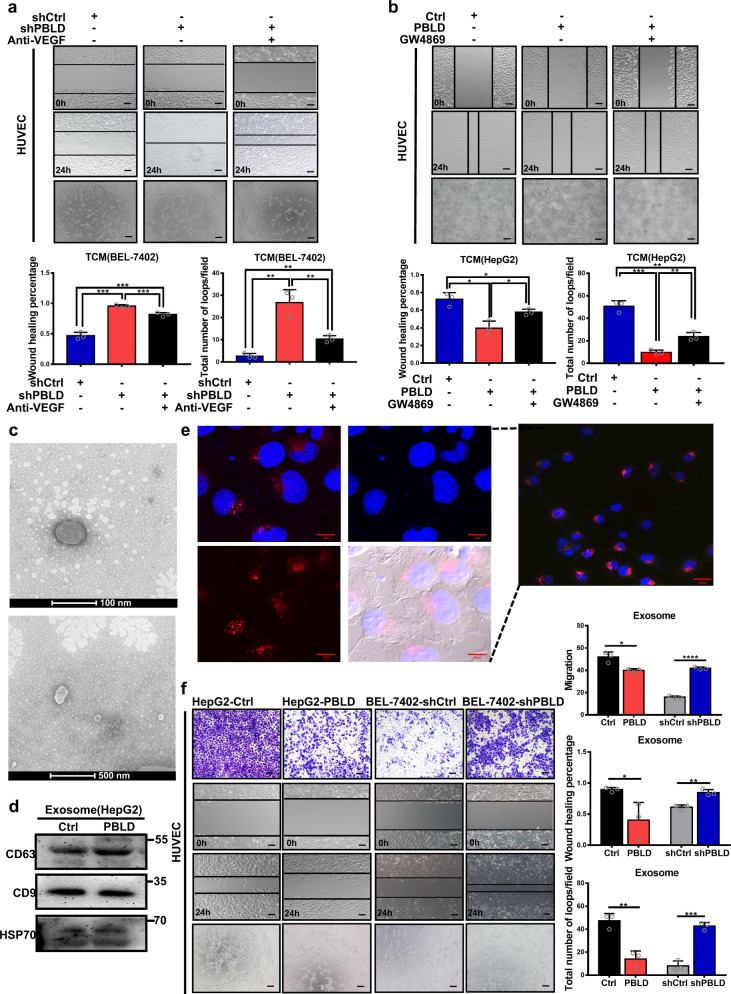
PBLD regulates angiogenesis through exosomes. **a** Migration and tube-formation abilities of HUVECs treated with TCM from BEL-7402-shPBLD and BEL-7402-shCtrl cells with VEGF antibody were detected by wound-healing and tube-formation assays. Scale bars, 200 µm. **b** Migration and tube-formation abilities of HUVECs cultured in TCM of HepG2-PBLD and HepG2-Ctrl cells pretreated with DMSO or GW4689 were detected by wound-healing and tube-formation assays. Scale bars, 200 µm. **c** Transmission electron microscopy micrograph of exosomes purified from HepG2 cell culture medium. **d** Expressions of specific exosomal markers, CD63, CD9 and HSP70 were identified by western blot. **e** The uptake of exosomes derived from HepG2 cells by HUVECs was observed and photographed under confocal microscopy. Red: exosomes stained with Dil; Blue: HUVECs nuclei stained with DAPI. **f** Migration and tube-formation abilities of HUVECs co-cultured with exosomes extracted from PBLD-upregulated and downregulated HCC cells were detected by Transwell, would-healing and tube-formation assays. *P < 0.05, **P < 0.01, ***P < 0.001, and ****P < 0.0001. Scale bars, 200 µm.

To verify the role of the HIF1-a/VEGF axis in PBLD-mediated inhibition of angiogenesis in HCC, we used VEGF neutralizing antibody to block secreted VEGF in HCC cells culture supernatant. Tube-formation and wound-healing assay showed that blocking VEGF secretion in BEL-7402-shPBLD partly decreased the PBLD-disruption induced promotion of tube-formation and migration ability of HUVECs. However, there were still significant differences compared with the shCtrl group (Fig. 4a). Thus, despite suppressing the HIF1-a/VEGF axis, there still exists HIF-1a/VEGF axis-independent pathway that regulates angiogenesis by PBLD. GW4869, an exosome release inhibitor, was used to identify whether exosomes contributed to the PBLD-regulated angiogenesis. HCC cells were pretreated with GW4869 (20 µM) for 48 h and the TCM was collected for the next 48 h. Then, the collected TCM was applied to culture HUVECs for 48 h. Inhibition of exosome secretion significantly attenuated PBLD induced suppression of HUVECs migration and tube-formation ability (Fig. 4b and Supplementary Fig. 4a, b). These results indicate that exosomes derived from HCC cells may also take part in the regulation of angiogenesis by PBLD.

Subsequently, the size and morphology of exosomes extracted from the culture supernatant of HCC cells were analyzed by transmission electron microscope (TEM). Results showed that isolated exosomes were of 50–100 nm diameter (Fig. 4c). Expression of specific exosomes markers, CD63, CD9, and HSP70, were also detected by western blot (Fig. 4d). The confocal images of the uptake of Dil-labeled tumor exosomes (Texo) by HUVECs were acquired (Fig. 4e). Further functional assays found that exosomes from HepG2-PBLD inhibited HUVECs migration and tube-formation abilities, contrary to exosomes derived from BEL-7402-shPBLD (Fig. 4f). Suppression of angiogenesis-related signaling pathway in HUVECs by exosomes derived from HepG2-PBLD was also confirmed by western blot (Supplementary Fig. 8d). Collectively, these data indicate that exosomes secreted from HCC cells also take part in PBLD-mediated regulation of angiogenesis.

### Exosomes from PBLD-overexpressed HCC cells have a high level of miR-940, which inhibits angiogenesis

**Fig. 5:**
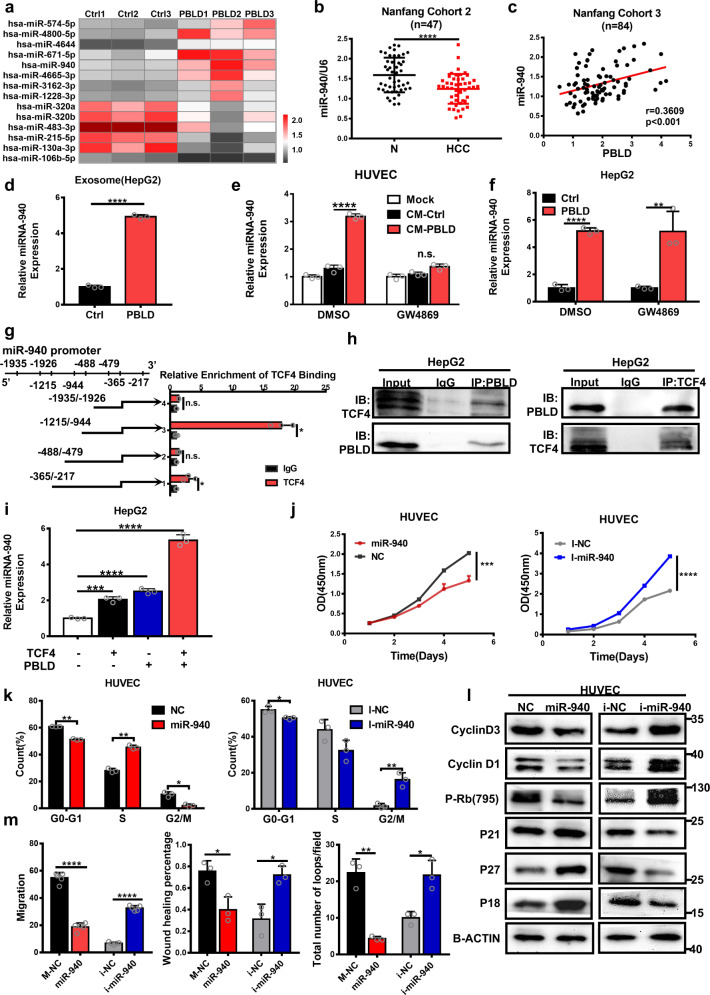
PBLD regulates angiogenesis through exosome-derived miR-940. **a** Hierarchical clustering of 14 differently expressed exosomal microRNA between exosomes from HepG2-PBLD and HepG2-ctrl group. **b** The expression of miR-940 was detected by qRT-PCR in 47 pairs of HCC tissue and the paired non-cancerous tissues (****P < 0.0001, paired *t*-test). **c** Spearman’s correlation analysis shows a positive correlation between PBLD and miR-940 levels in 84 HCC tissue samples from Nanfang cohort 3 (*P* < 0.001, *r* = 0.3609). **d** The expression of miR-940 was detected by qRT-PCR in exosome extracted from HepG2-PBLD and HepG2-Ctrl groups. **e** Detection of miR-940 expression by qRT-PCR in HepG2-NC and HepG2-PBLD treated with DMSO or GW4689. **f** shows the detection of miR-940 expression by qRT-PCR in HUVECs that cultured in TCM of HepG2-NC and HepG2-PBLD pretreated with DMSO or GW4689. **g** ChIP-qPCR analysis of TCF4-binding regions in miR-940 promoter shows the important sites including-365/-217, -1215/-944 in HepG2 cells. **h** The interaction of TCF4 with PBLD is shown by co-immunoprecipitation assay using TCF4 and PBLD antibodies in HepG2 cells. **i** MiR-940 expression in HepG2 cells transfected with PBLD and TCF4 plasmids either together or separately. **j** The growth curve of HUVECs transfected with miR-940 mimics and inhibitors were obtained by the CCK-8 assay. **k** Cell cycle analysis showed that miR-940-induced cell cycle arrest and miR-940 inhibitors could accelerate the transition in HUVECs. **l** Expression of cell cycle-related proteins detected by western blot in HUVECs transfected with miR-940 mimics and inhibitors. **m** The migration and tube-formation ability of HUVECs transfected with miR-940 mimics and inhibitors was detected by Transwell, wound-healing and tube-formation assays. *P < 0.05, **P < 0.01, ***P < 0.001, and ****P < 0.0001.

To clarify the mechanism underlying the PBLD regulation of angiogenesis by exosomes, microRNA microarray was performed to examine the differential expression profile of PBLD-related microRNA in HCC exosomes. According to the foldchange and *P* value (foldchange >3, *P* < 0.05), 14 differentially expressed microRNAs were discovered (Fig. 5a and Supplementary Table 4). After qRT-PCR verification in 47 pairs of HCC fresh samples, miR-940 was found significantly downregulated in HCC tissues compared with those in matched normal tissues (Fig. 5b). Moreover, qRT-PCR conducted on 84 HCC tissues also revealed a positive correlation between the expression of miR-940 and PBLD (Fig. 5c). The expression of miR-940 in PBLD-upregulated HCC cells, secreted exosomes and co-cultured HUVECs were all clearly elevated compared with those in control groups (Fig. 5d–f). Inhibition of HCC cells exosome secretion using GW4869 disrupted this difference in miR-940 expression between the two groups of HUVECs (Fig. 5e, f).

Furthermore, we explored the mechanism of PBLD action in regulating the expression of miR-940. First, we use two online databases, PROMO (http://alggen.lsi.upc.es/cgi-bin/promo_v3/promo/promoinit.cgi?dirDB=TF_8.3) and Transmir (http://www.cuilab.cn/transmir) to predict the transcription factors for miR-940. Next, three online databases (BioGRID, HitPredict, GPS-Prot) for protein–protein interaction were used to screen the protein interacting with PBLD (Supplementary Fig. 5a–d). According to the two-way Venn diagram (Supplementary Fig. 5a), TCF4 was the only gene predicted to be the transcription factor of miR-940 that also interacted with PBLD. The potential TCF4-binding sites along the miR-940 promoter region were predicted by JASPAR (http://jaspar.genereg.net/). Interestingly, compared to overexpression of TCF4 or PBLD separately, their simultaneous co-expression induced a dramatic upregulation of miR-940 (Fig. 5i). Several transcriptional regulators have been proved to interact with TCF4, thus, affecting its transactivation activity. Here, a direct interaction of PBLD with TCF4 was observed through co-immunoprecipitation assay in HepG2 cells (Fig. 5h). Then, TCF4 binding to the promoter region of miR-940 was identified by chromatin immunoprecipitation (ChIP)-qPCR analysis. Fragments of the miR-940 promoters were detected by qPCR to verify the potential TCF4-binding sites. Among these predicted recognition sites, two (–365/–217, –1215/–944) were specifically enriched by anti-TCF4 antibody compared with the IgG control (Fig. 5g). Thus, PBLD interacts directly with TCF4 and activates TCF4-mediated transcriptional promotion of miR-940.

Next, we investigated whether miR-940 played the same role with the HCC-derived exosomes in HUVECs. CCK-8 and cell cycle assay revealed that miR-940 suppressed HUVECs growth and cell cycle transition (Fig. 5j, k). Accordingly, the level of cell cycle-associated proteins also changed, miR-940-mimics induced upregulation of p18, P21, and P27, and downregulation of P-Rb (795), Cyclin D1 and Cyclin D3. HUVECs transfected with miR-940 inhibitors showed a reverse trend (Fig. 5l and Supplementary Fig. 6b). Similarly, miR-940 inhibited HUVECs migration and tube formation (Fig. 5m and Supplementary Fig. 6a). These results suggest that exosomal miR-940 participates in PBLD-mediated regulation of angiogenesis.

### MiR-940 regulates the expression of VEGFR2 by targeting ETS1 in HUVECs

**Fig. 6:**
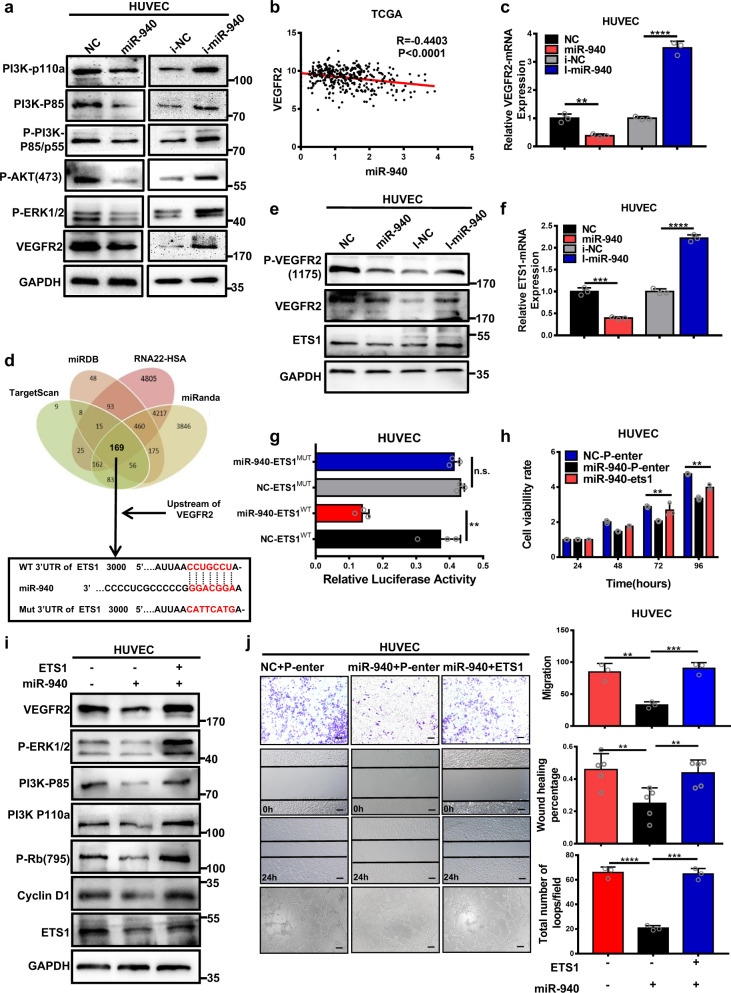
MiR-940 downregulates the expression of VEGFR2 by targeting ETS1 in HUVECs. **a** Results of western blot show the effects of miR-940 on VEGFR2 expression and the signaling pathways downstream of VEGF/VEGFR2 in HUVECs. **b** Analysis of HCC related sequencing data from the TCGA database reveals a negative correlation of miR-940 with VEGFR2 (*P* < 0.0001, *r* = –0.4403, *n* = 366, Spearman’s correlation analysis). **c** The relative mRNA expression of VEGFR2 in HUVECs cell transfected with miR-940 mimics or inhibitor. **d** The four-way Venn diagram indicating the number of overlapping genes in four public miRNA target prediction databases (TargetScan, miRanda, miRDB, RNA22-HSA). Combined with the upstream of VEGFR2, ETS1 was screened out to be the possible target gene for miR-940. Bioinformatics analysis to reveal the 3′UTR region of ETS1 containing miR-940 binding sites, and the seed region is highlighted. **e** Western blot detected ETS1, P-VEGFR2 (1175) and VEGFR2 protein expression in HUVECs transfected with miR-940 mimics or inhibitors. **f** The relative mRNA expression of ETS1 in HUVECs transfected with miR-940 mimics or inhibitors. **g** Dual-luciferase reporter assay verified the binding of miR-940 on the 3′UTR region of ETS1 in HUVECs. **h** The proliferation ability of HUVECs was detected by CCK-8 assay, and ETS1 could reverse the miR-940 induced suppression of cell growth. **i** Western blot analysis of ETS1, VEGFR2, and the signaling pathways downstream of VEGF/VEGFR2 in HUVECs co-transfected with miR-940 mimics and ETS1 plasmids. **j** The migration and tube-formation ability of HUVECs co-transfected with miR-940 mimics and ETS1 plasmids was detected by Transwell, wound-healing and tube-formation assay. *P < 0.05, **P < 0.01, ***P < 0.001, and ****P < 0.0001. Scale bars, 200 µm.

VEGF exerts pro-angiogenic effects mainly by binding to VEGFR2. Therefore, we further investigated whether miR-940-mediated suppression in angiogenesis was achieved through VEGFR2. TCGA database analysis revealed a negative correlation between miR-940 and VEGFR2 expression (Fig. 6b). Further verification using western blot and qRT-PCR reconfirmed the negative regulation of VEGFR2 expression and signaling pathways downstream of VEGF/VEGFR by miR-940 (Fig. 6a, c and Supplementary Fig. 6c). In addition, miR-940 could also inhibit VEGF-stimulated angiogenesis in HUVECs. Compared to the HUVECs transfected with miR-940 mimics, NC groups showed a more significant increase in migration and tube-formation ability after VEGF treatment, contrary to miR-940 inhibitors (Supplementary Fig. 7a, b). Sorafenib is a first-line treatment for advanced HCC, which acts as a VEGFR2 inhibitor. MiR-940 was found to promote HUVECs apoptosis after sorafenib treatment, indicating enhanced sorafenib sensitivity (Supplementary Fig. 7d). The level of cell apoptotic protein (cleaved-caspase 3, 7, 9) also changed accordingly (Supplementary Fig. 7c). These results indicate that miR-940 inhibits angiogenesis through VEGFR2.

To explore the regulatory mechanism of miR-940 on VEGFR2, online microRNA target prediction databases (TargetScan, miRanda, miRDB, RNA22-HSA) were used to predict the miR-940 target genes (Fig. 6d). Next, four predicted gene sets were intersected with possible upstream regulators of VEGFR2. Finally, ETS1, an important transcription factor of VEGFR2, was found to be a candidate target gene of miR-940. Negative regulation of ETS1 by miR-940 was verified in HCC cells and the TCGA database (Fig. 6e, f and Supplementary Fig. 6d). Furthermore, the complementary site for the seed region of miR-940 in the 3’UTR of ETS1 mRNA and their mutant segments were cloned to construct the dual-luciferase reporter plasmids. The results showed reduced luciferase activity of WT ETS1 reporter gene when co-transfected with miR-940 mimics (Fig. 6g). In general, these data confirm that ETS1 is the direct target of miR-940. We also found that miR-940-induced suppression of proliferation, migration and tube-formation ability of HUVEC were recovered by rescuing ETS1 expression (Fig. 6h, j). Moreover, western blot revealed that the negative regulation of VEGFR2 and related downstream genes expression could be reversed by ETS1 (Fig. 6i and Supplementary Fig. 6e). Thus, miR-940 mediates negative effects on angiogenesis by targeting the ETS1/VEGFR2 axis.

### PBLD inhibits angiogenesis through exosomes/miR-940/ETS1 axis

**Fig. 7:**
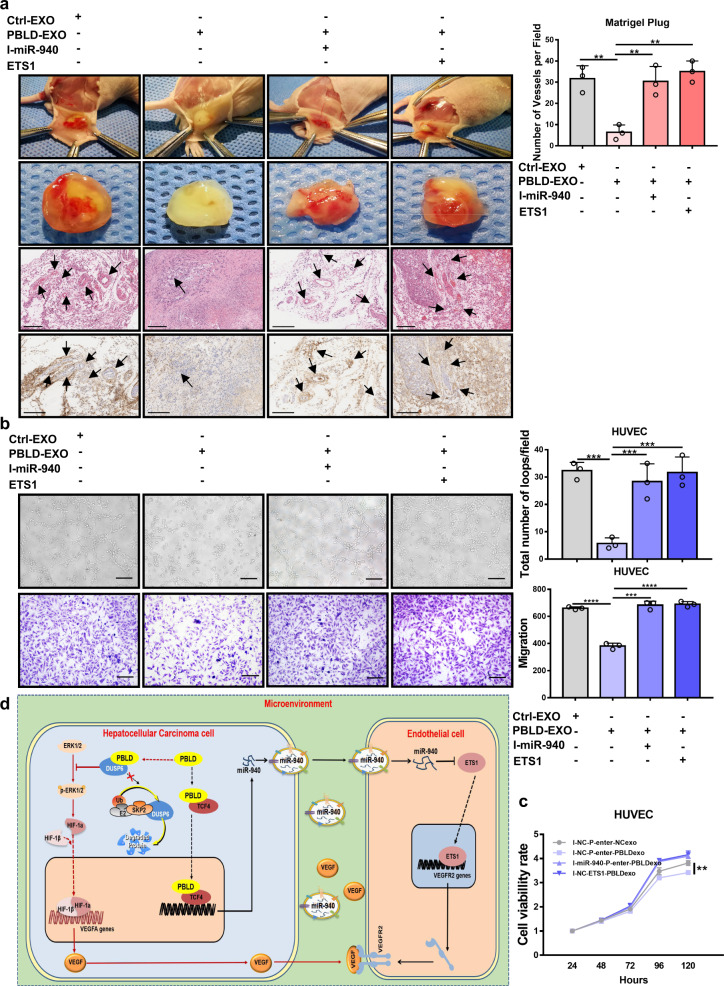
The exosome/miR-940/ETS1 axis takes part in the regulation of angiogenesis by PBLD. **a** In vivo Matrigel plug assay. HUVECs were treated differently according to the corresponding groups: HUVEC^i-NC/p-Enter^+Exo-Ctrl, HUVEC^i-NC/p-Enter^+Exo-PBLD, HUVEC^i-miR-940/p-Enter^+Exo-PBLD, HUVEC^i-NC/ETS1^+Exo-PBLD. As shown in the figure, from top to bottom: the angiogenesis of Matrigel plugs capsule; gross morphology of Matrigel plugs; paraffin sections of formalin-fixed Matrigel plugs were stained with hematoxylin and eosin (H&E), and the black arrows indicate microvessel; CD31 immunohistochemical staining of paraffin sections of formalin-fixed Matrigel plugs. Scale bars, 200 µm. The numbers of vessels per field inside the Matrigel plug was quantified by H&E staining. **b**, **c** HUVECs were divided into four groups: HUVEC^i-NC/p-Enter^+Exo-Ctrl, HUVEC^i-NC/p-Enter^+Exo-PBLD, HUVEC^i-miR-940/p-Enter^+Exo-PBLD, HUVEC^i-NC/ETS1^+Exo-PBLD. Then, proliferation ability, migration and tube-formation ability were detected by CCK-8, transwell and tube-formation assay in four groups HUVECs, Scale bars, 200 µm. **d** A hypothesized signaling mechanism of PBLD-mediated suppression of angiogenesis in HCC. *P < 0.05, **P < 0.01, ***P < 0.001, and ****P < 0.0001, n.s. non-significant.

Next, we evaluated the effects of exosomes/miR-940/ETS1 axis on PBLD-regulated angiogenesis. HUVECs co-cultured with exosomes derived from HepG2-PBLD (Exo-PBLD group) had a decreased tube-formation, proliferation, and migration abilities in comparison to the control group. However, blocking the expression of miR-940 or upregulation of ETS1 in HUVECs, by pretreating HUVECs with miR-940 inhibitors (940-inhibitor group) and ETS1 plasmids (ETS1 group), could abolish the effects of exosomes on HUVECs (Fig. 7b, c). We also observed the same phenomenon in the Matrigel plug assay in vivo. The density of vessels on the capsule of the Matrigel plugin Exo-PBLD group was lower than that in the control groups, and hematoxylin and eosin (H&E) staining and CD31 immunohistochemical staining showed lower MVD inside the Matrigel plug of Exo-PBLD group. While 940-inhibitor group and ETS1 group showed higher MVD on either surface (inside and outside) of the Matrigel plug (Fig. 7a and Supplementary Fig. 8b, c).

## Discussion

In this study, we demonstrate that PBLD functions as a suppressor of angiogenesis in HCC by modulating the molecular regulatory network in TME. We first found a negative correlation between PBLD and a series of angiogenesis-related genes, including VEGF and HIF-1a, by analyzing the TCGA database and our previous microarray data. Second, compared with the control group, TCM from PBLD-overexpressed HCC cells inhibited proliferative, migratory, and tube-formation abilities of HUVECs. This negative regulation of angiogenesis was partly due to a lower expression and secretion level of VEGF induced by PBLD overexpression through the ERK/HIF-1a/VEGF axis in HCC cells. PBLD was also found to be negatively associated with the expression of CD31 and HIF-1a in HCC samples. Third, exosomal miR-940 transmitted from PBLD-overexpressed HCC cells suppressed angiogenesis through the ETS1/VEGFR2 axis in HUVECs. These findings might provide a new potential target for anti-angiogenesis therapy in HCC.

Relatively limited benefits and development of resistance significantly restrict anti-angiogenesis therapy extensive application [2]. Hypoxia is one of the most widely accepted mechanisms underlying the resistance to anti-angiogenesis therapy. Sustained anti-angiogenesis therapy increases the level of tumor hypoxia, leading to increased expression of HIF-1a, which promotes the production of pro-angiogenesis genes, thereby contributing to the resistance of HCC cells to anti-angiogenesis therapy [7]. Liang et al. observed increased HIF-1a expression appeared in patients receiving sorafenib treatment compared to those without sorafenib treatment, and a higher HIF-1a expression was observed in sorafenib resistant patients than in sensitive patients [19]. Here, we found that PBLD exerted anti-angiogenic effects by blocking VEGFR2 expression on HUVECs while downregulating HIF-1a-induced VEGF expression and secretion in HCC cells, which may contribute to reduce the hypoxia-induced resistance to anti-angiogenesis therapy. That indicates that PBLD blocks angiogenesis while avoiding the increased HIF-1a expression in cancer cells resulted from anti-angiogenesis induced hypoxia. Thereby, our research may provide a therapeutic target and strategy for overcoming resistance to anti-angiogenesis therapy.

HIF-1a, the core transcriptional regulator during hypoxia response, undergoes degradation via a pVHL-dependent pathway under normoxic conditions [20]. While under hypoxic conditions, HIF-1a dimerizes with HIF-1b and translocate into the nucleus and acts on gene transcription [21]. Besides this oxygen level-dependent regulation, expression and transcriptional activity of HIF-1a are also controlled by post-transcriptional modification [22]. Activation of the ERK/MAPK pathway increases the synthesis of HIF-1a protein by promoting its mRNA translation. Moreover, activated ERK promotes nuclear retention of HIF-1a, thereby stimulating its transcriptional promoting functions [23]. Here, we verified that the expression and nuclear translocation of HIF-1a were regulated by PBLD. Moreover, GSEA analysis of TCGA, GSE116174 datasets and our previous microarray data indicated that PBLD expression was associated with ERK/MAPK signaling pathway. Follow-up ERK/MAPK inhibitor and activator experiments confirmed that PBLD regulated the HIF-1a/VEGF axis via the ERK/MAPK pathway.

ERK pathway, which is one of the most widely studied MAPK pathways, plays a key role in diverse cellular pathological processes including cancer [24]. Spatiotemporal specificity, intensity and duration of MAPK activity are strictly regulated by dual-specificity MAP kinase phosphatases (MKPs) [25]. DUSP6 is an ERK-specific MKP that is located in the cytoplasm. In our study, we found that PBLD positively regulated DUSP6 expression, while no significant change was observed in the mRNA level of DUSP6. Restoration of DUSP6 expression suppressed the PBLD-knockdown induced activation of ERK phosphorylation. These results indicate that PBLD might manipulate ERK phosphorylation via post-transcriptional regulation of DUSP6. Post-translational modification including ubiquitination is a critical mechanism in regulating DUSP6 stability and activity [26]. PKCδ depletion promotes ubiquitination and degradation of DUSP6 by upregulating ubiquitin E3 ligase Nedd4, thus increasing ERK phosphorylation [27]. Similarly, metformin activates ERK/MAPK by enhancing B-Raf (V600E) induced DUSP6 degradation, thus promoting melanoma progression [28]. Here, we found that PBLD prolonged the DUSP6 half-life and decreased its ubiquitination. We also identified the ubiquitin E3 ligase SKP2 contributed to the ubiquitination of DUSP6 in HCC cells. Finally, PBLD could maintain DUSP6 expression by competitively binding to it, and inhibiting SKP2-induced DUSP6 degradation.

MicroRNAs carried in Texo regulate tumor angiogenesis via post-transcriptional negative regulation of target genes [29, 30]. Exosomal miR-451a derived from HCC cells inhibit proliferation, migration and angiogenesis of endothelial cells [31]. Another study in breast cancer finds that docosahexaenoic acid mediates the anti-angiogenesis effect through targeting a variety of angiogenesis-related genes by exosomal miR-23b and miR-320b from cancer cells [32]. In our study, miR-940 was found upregulated in exosomes derived from PBLD-overexpressed HCC cells according to the microarray data. Subsequent validation in clinical samples reconfirmed the positive correlation between PBLD and miR-940 expression. MiR-940 is closely related to the progression of various human cancers including HCC. Mechanically, miR-940 serves as a tumor suppressor via targeting genes associated with tumor progression including H1HR and SPOCK1 in HCC [33, 34]. Besides, miR-940 also takes part in intercellular communication in TME. An ovarian cancer study revealed that miR-940 carried in tumor cells exosomes promotes M2-type polarization of tumor-associated macrophages [35]. However, the molecular function of miR-940 in HCC angiogenesis still remains to be explored. Here, we found that exosomal miR-940 derived from HCC cells participated in the PBLD-mediated regulation of angiogenesis. Further exploration revealed that miR-940 repressed the proliferative, migratory and tube-formation ability of HUVECs through targeting the ETS1/VEGFR2 axis [36]. In our study, ETS1 was identified as a target gene of miR-940 and contributed to the miR-940 mediated suppression of angiogenesis. These results suggest that miR-940 might be a promising angiogenic signaling candidate in the progression of HCC.

We also explored the mechanism of miR-940 regulation by PBLD. Through bioinformatics analysis, TCF4 was predicted to interact with PBLD and also has four binding sites on the miR-940 promoter region. TCF4, a basic helix-loop-helix transcription factor, shows especially high expression levels in the embryonic central nervous system, mesoderm and adult brain and plays a complex role in a variety of developmental processes and disorders [37]. The role of TCF4 act as a transcriptional repressor or a promoter is highly context-dependent. These contrasting effects on transcription are mainly due to the specific interaction with transcriptional regulators via its modular domain structure [38]. One of the TCF4 activation domains (AD1) is confirmed to bind with EP300, a transcriptional activator, thus activating TCF4 transcriptional promotion effects [39]. Moreover, runt-related transcription factor 1 also competes with EP300 for binding with AD1, thereby attenuating the transcriptional activity of TCF4 [40]. Here, we found that TCF4 interacted with PBLD and participated in the PBLD-mediated negative regulation of angiogenesis by promoting miR-940 transcription. This may provide evidence for the regulatory role of TCF4 in angiogenesis.

In conclusion, we identified PBLD as a key molecular in remolding the tumor angiogenic microenvironment, and elucidated the molecular regulatory network involved in PBLD-mediated suppression of HCC angiogenesis. The DUSP6-ERK-HIF-1a-VEGF regulatory axis was demonstrated to function in PBLD-mediated decrease in VEGF expression and secretion in HCC cells, and PBLD-mediated downregulation of VEGFR2 in HUVECs was through the Texo-miR-940-EST1-VEGFR2 axis (Fig. 7d). This study will contribute to a more in-depth understanding of the complex regulatory network between TME and angiogenesis in HCC, and thus may provide new insight into the tumor anti-angiogenesis and combination strategies.

## Materials and methods

### Clinical specimens

Tumor tissues, normal adjacent tissues of patients with pathologically diagnosed HCC were collected from Nanfang Hospital, Southern Medical University. The study was approved by the ethics committee of the Nanfang Hospital, and informed consent was obtained from each patient.

### Cell lines

Immortalized HCC cell lines HepG2 and HUVEC were obtained from American Type Culture Collection (Manassas, VA, USA). Immortalized HCC cell lines BEL-7402 were obtained from Cell Bank of China Science Academy (Shanghai, China). All cell lines used in this study were tested by short tandem repeat analysis, validated to be free of mycoplasma. Cells were cultured in DMEM (Gibco, USA) or RPM1640 medium supplemented with 10% fetal bovine serum (BI, Israel), maintained at 37 °C, in a 5% CO_2_ incubator, and used within 30 passages for all experiments.

### Reagents

Sorafenib (MCE, USA) was dissolved in DMSO, the stock solutions (90 mM) were stored at –80 °C in single-dose vials, avoiding repeated freeze-thaw. Dilute the stock solutions with PBS to a working Solution (90 nM) for inhibiting VEGFR2 according to the user instruction. GW4869 (MCE, USA) was stored at −80 °C as a 1.5 mM stock suspension in DMSO, avoiding repeated freeze-thaw. Dilute the stock solutions with PBS to a concentration of 20 μM for inhibition of exosome release. CHX (Selleck, USA) was dissolved in DMSO and stored at –80 °C in single-dose vials and diluted to a working solution (20 μg/ml) for inhibiting protein synthesis. MG132 (MCE, USA) was dissolved in DMSO and stored at –80 °C in the stock solutions(10 mM), diluted to a working Solution (10 μM) for inhibiting the proteolytic activity of the 26S proteasome complex. U0126 (Selleck, USA) was dissolved in DMSO and diluted to a working Solution (10 μM) for inhibiting MEK1 and MEK2. PMA (Selleck, USA) was dissolved in DMSO and diluted to a working solution (100 nM) for activating ERK phosphorylation.

### Lentivirus transduction, oligonucleotides, and plasmid transfections

PBLD overexpressed and interfered lentivirus were synthesized by Genechem Co., Ltd. (Shanghai, China). The cells were infected with lentivirus at different multiplicity of infection, and were detected by qRT-PCR, then select the optimal experimental conditions. The miR-940 mimics, negative control (NC), miR-940-inhibitor, and i-NC were purchased from GenePharma (Shanghai, China). DUSP6-WT, DUSP6-MUT (K68R, K254R, K324R, K327R), ETS1, SKP2, and TCF4 plasmids were purchased from Vigenebio (Shandong, China). These oligonucleotides and plasmids were transfected in serial concentration gradient together with Lipofectamine 3000 (Invitrogen, USA), then qRT-PCR or western blot was used to detect the optimization concentration.

### RNA isolation, quantitative real-time PCR, and microarray analysis

RNA isolation, quantitative real-time PCR, and microarray analysis were conducted as previously described [41]. See in Supplementary Materials and Methods for additional details. The primers used in this study are listed in Supplementary Table 5. The differentially expressed microRNAs detected by microarray analysis are shown in Supplementary Table 4. We had also deposited our microarray data that supported the findings of our research in a public repository (Gene Expression Omnibus), Series GSE185879.

### Tissue microarray, immunohistochemistry, immunofluorescence, and western blot assay

Tissue microarray containing 90 pairs of HCC tissues and their matched normal tissues was obtained from Shanghai Outdo Biotech. Co., Ltd (Shanghai, China). The immunohistochemistry, immunofluorescence, and western blot assay were conducted as previously described [42, 43]. See in Supplementary Materials and Methods for additional details. The primary and secondary antibodies used in this study are listed in Supplementary Table 6.

### Migration and cell count kit-8 assay

CCK-8 assay and migration assay, including Transwell and wound-healing assay, were conducted as previously described [41]. See in Supplementary Materials and Methods for additional details.

### Apoptosis and cell cycle assays

Apoptosis and cell cycle assays were conducted as previously described [41], using propidium iodide (BD, USA) and PE Annexin V Apoptosis Detection Kit (BD, USA). See in Supplementary Materials and Methods for additional details.

### Tube-formation assay

In all, 50 µL Matrigel (BD Biosciences, USA) was placed into 96-well plates and kept at 37 °C for 1 h. Cell suspensions in culture medium containing 1.5 × 10^4^ HUVECs were seeded in wells coated with Matrigel in advance. The plate was cultured in humidified 37 °C, 5% CO_2_ for 2 h. Then, the number of tube-like structures was visualized by microscopy, and analyzed by image-Pro Plus software.

### Isolation of exosomes

For exosomes isolation, HCC cells were seeded in sterile cell culture dishes. When cell confluency reached over 60%, the original culture medium was replaced by exosome-depleted FBS (SBI, USA) medium. After 48 h, the cell culture medium was filtered with a 0.22 µm filter (Millipore, USA) and collected for exosome isolation using ExoQuick-TC^®^ ULTRA kit (SBI, USA). In all, 1 mL of ExoQuick-TC reagent was added to 5 mL of cell culture medium and maintained at 4 °C overnight. Then, the exosome precipitate was harvested by centrifugation according to the manufacturer’s instructions. The isolated exosomes were resuspended by PBS and stored at –80 °C for further use. The concentration of exosome suspensions was measured using the BCA method and AchE assay.

### Identification and labeling of exosomes

The expression of exosome surface marker was detected by western blot using CD63, CD9 and Hsp70 antibodies (1:1000, SBI, USA). After appropriate specimen processing, the morphology and diameter of exosomes were observed and estimated by TEM. The isolated exosomes were labeled using Dil. Exosomes marked with Dil were co-cultured with HUVEC at 37 °C, in 5% CO_2_ for 1 h. Then the cells were fixed with 4% paraformaldehyde, and the endocytosis of labeled exosomes was visualized by confocal laser scanning microscope (Olympus-FV1200, Olympus, Japan).

### Luciferase reporter assay

This assay was performed as previously described [41], using Dual-Luciferase^®^ Reporter (DLR™) Assay Kit (Promega, USA). See in Supplementary Materials and Methods for additional details.

### Co-immunoprecipitation

Co-IP assay was conducted on 1 × 10^7^ HCC cells. After being washed with pre-chilled PBS twice, the cells were scraped and lysed with cold NP-40 premixed with protease and phosphatase inhibitors. Total protein extract was collected after 12,000 rpm centrifugation. In all, 25 μL protein A/G magnetic beads (MCE, USA) for each IP test were pre-incubated with diluted antibody (2 ug Ab/test) 2 h at 4 °C. Then, the magnetic beads-Ab complex was harvested via magnetic separation. Then, our prepared sample was mixed with the magnetic beads-Ab complex and incubated at 4 °C overnight. Finally, the immunoprecipitates were collected after magnetic beads elution. Following that, western blot assay was performed to verify the interaction of proteins. We used anti-rabbit IgG as an NC.

### Chromatin immunoprecipitation assay

ChIP assay was conducted using simpleChIP™ Enzymatic Chromatin IP Kit (CST, USA) in HCC cells transfected with TCF4-p-Enter plasmids for 48 h. Rabbit monoclonal anti-TCF4 antibody (Abcam, USA) was used for immunoprecipitation according to the manufacturer’s instructions. To further confirm the TCF4-binding sites in the promoter of miR-940, four pairs of specific primers were used for qRT-PCR analysis.

### In vivo Matrigel plug assay

Male BALB/c nude mice aged 6–7 weeks were purchased from the Central Laboratory of Animal Science at Southern Medical University, and maintained at the Laboratory Animal Centre of Nanfang Hospital in a specific pathogen-free environment. They were randomly assigned to different groups, and each group have at least three mice. HUVECs were transfected with miR-940-inhibitor, i-NC, ETS1 and p-Enter plasmid according to the corresponding group. In all, 200 µL pretreated HUVECs suspension (5 × 10^6^ cells) were mixed with 300 µL Matrigel (BD Biosciences, USA) and exosomes derived from HCC cells. Next, the Matrigel plug was injected subcutaneously in the right dorsal region of the nude mice. On day 8, Matrigel plugs were harvested and the density of vessels on the capsule of Matrigel plug was calculated. Then, the samples were fixed in 10% formaldehyde and embedded in paraffin for further immunohistochemistry and H&E staining. Five random microscopic fields of each sample were considered for quantitative analysis of MVD inside the Matrigel plugs. All mouse experiments were approved by the Nanfang Hospital Animal Ethics Committee.

### Statistical analysis

Data collected from at least three independent repeated experiments were presented in the form of mean ± SD. The SPSS 25.0 (IBM; Chicago, IL, USA) and GraphPad Prism 7.0 were used to perform statistical analyses. The significant difference between the two groups was measured by Student’s *t*-test. Paired sample *t*-test was used to measure the expression difference between HCC and matched normal tissues. Spearman’s rank correlation test was used to measure the bivariate correlations between the expression of genes. The Kaplan–Meier method was used to plot survival curves. *P < 0.05, **P < 0.01, ***P < 0.001, and ****P < 0.0001 were considered statistically significant.

## Supplementary information

Supplementary information

## Data Availability

The datasets generated and analyzed during the current study are available from the corresponding author on reasonable request.
