# Supplementary material for: PBLD inhibits angiogenesis via impeding VEGF/VEGFR2-mediated microenvironmental cross-talk between HCC cells and endothelial cells
Source: Oncogene. 2022 Feb 10;41(13):1851–65. doi: 10.1038/s41388-022-02197-x (PMC8956508; doi:10.1038/s41388-022-02197-x)
Supplement: Supplementary file 1 — Supplementary information [file 41388_2022_2197_MOESM1_ESM.pdf]

Supplementary information summary

1. **Supplementary material and methods**
2. **Supplementary figure 1 (PDF)**

   PBLD inhibits angiogenesis induced by hypoxia
3. **Supplementary figure 2 (PDF)**

   PBLD suppresses HIF1-a/VEGF axis via ERK/MAPK signaling pathway
4. **Supplementary figure 3 (PDF)**

   PBLD decreases ERK1/2 phosphorylation by protecting DUSP6 from ubiquitin-proteasome degradation
5. **Supplementary figure 4 (PDF)**

   PBLD-mediated regulation of angiogenesis through exosomes
6. **Supplementary figure 5 (PDF)**

   PBLD regulates miR-940 expression by interacting with TCF4
7. **Supplementary figure 6 (PDF)**

   MiR-940 suppresses VEGF/VEGFR2 signaling pathway
8. **Supplementary figure 7 (PDF)**

   MiR-940 inhibits angiogenesis through regulation of VEGFR2
9. **Supplementary figure 8 (PDF)**

   PBLD inhibits angiogenesis via exosome/miR-940/ETS1 axis
10. **Supplementary table 1 (PDF)**

    PBLD IHC score of HCC tissues and their matched normal tissues
11. **Supplementary table 2 (PDF)**

HIF-1a IHC score of HCC tissues

12. **Supplementary table 3 (PDF)**

    Predicted E3 ligases for DUSP6

13. **Supplementary table 4 (PDF)**

    List of upregulated microRNAs in exosome from HepG2 cells by comparing the PBLD-overexpression group and the control group

14. **Supplementary table 5 (PDF)**

    The sequences of PCR primers and siRNAs

15. **Supplementary table 6 (PDF)**

    List of primary and secondary antibodies used in the study

16. **Supplementary table 7 (PDF)**

    Clinicopathologic features of patients in Nanfang cohort

Supplementary material and methods

RNA isolation, quantitative real-time PCR

Total RNA of clinical tissue and HCC cells was extracted by Trizol reagent (Takara, USA). Quantity and quality of extracted total RNA were assessed by NanoDrop2000 ultra-micro spectrophotometer. PrimeScript RT reagent Kit(Takara, USA) and SYBR Premix Ex Taq (Takara, USA) were used to synthesize cDNA and quantitative real-time PCR(qRT-PCR) of mRNA, GAPDH was used as a reference gene. Mir-XTM miRNA First-Strand Synthesis Kit (Takara, USA) and SYBR Premix Ex Taq (Takara, USA) were used to synthesize cDNA and qRT-PCR of microRNA, U6 was used as internal control for normalization. 2–ΔΔCt method was used to perform analysis of qRT-PCR results. The primer sequences are shown in Supplementary Table 5.

Microarray assay

Extracted total RNA of HepG2 Cells -derived exosomes from PBLD-overexpression group and the control group were processed following the standard Agilent protocol, and each group has three biological replicated samples. Agilent human miRNA array detection and data normalization were conducted by Shanghai Biotechnology Corporation. Part of differentially expressed microRNAs were shown in Supplementary Table 4.

Western blot assay

Total protein of tissue and cells was extracted using mixture of RIPA Lysis Buffer (medium, Beyotime, China) and PMSF (Fdbio science, China). The concentration of the protein samples was determined by BCA Protein Assay Kits (Beyotime, China). Then, loading protein samples and appropriate protein markers into the SDS-PAGE gel. After electrophoresis and electrotransferred to PVDF(0.45μm, Thermo, USA) membrane, 5% skim milk was used to block the protein bands, and next

incubated with primary antibody overnight at 4°C on a benchtop rocker. Following the incubation with a suitable HRP-conjugated secondary antibody, the protein bands were soaked in prepared ECL substrate and visualized by chemiluminescence imaging system. Finally, quantitative analysis of target protein expression was done by calculating of protein bands gray values. Primary and secondary antibodies used in this assay were listed in Supplementary Table 6.

Cell count kit-8 assay

After cell number determination, cells were seeded in a 96-well plate at a density of 1000 cells/well with 100μL of culture medium and cultured at 37°C, in 5% CO_2 incubator overnight. 10μL CCK-8 solution (Dojindo, Japan) were added to each testing well of the plate and incubated for 2 h in the incubator. Then, the absorbance was measure at 450 nm using Paradigm Detection Platform((Beckman, CA, USA). Continuous detections were performed during the following 4 days at the same time point, and growth curve was plotted after the final detection.

Migration assays

For the wound healing assay, HUVEC cells were seeded into six-well plates and cultured until confluent. Using a pipette tip to make a cross scratch on the center of each well, mimicing a wound. Then, the wells were replenished with serum-free medium and kept in 37°C, in 5% CO_2 incubator. Gap distance of wound in each well was photographed and recorded every 12 hours. For the Transwell migration assay, HUVEC cells were resuspended with FBS free medium at a concentration of 1 x10^5 cells/ml. 0.2ml cell suspension was added into Transwell insert, while the lower chamber was filled with 0.6ml medium including 10% FBS. After 24 h incubation, the cells inside of the Transwell insert were removed, the cells outside the Transwell insert were fixed and stained with crystal violet for the following analysis.

Apoptosis and cell cycle assays

For cell cycle assay, single cell suspension was prepared to be fixed with 70% ethanol at 4°C overnight, then incubated with DNA fluorescent dye Propidium Iodide (BD, USA) at a cell density of 5 $x10^5$ to $1x10^6$ cells/mL for 30 min at room temperature (RT). The content of DNA was determined by flow cytometry, and revealed the cell distribution of four sequential phases in cell cycle. For apoptosis assay, properly treated cells were incubated with 5 µl of PE Annexin V and 5 µl 7-AAD at a concentration of 1 x 10^6 cells/ml for 15 min at room temperature in the dark. The percentage of cells that were undergoing apoptosis were quantitatively determine by flow cytometry following the PE Annexin V Apoptosis Detection Kit (BD, USA) protocol.

Immunohistochemistry

After paraffin embedding, the tissues were continually sectioned into 4µm thin slices. The section underwent dewaxing in xylene and rehydration in graded concentrations of ethanol. High pressure method was taken to perform antigen retrieval. The slides were blocked with 10% serum of the species from which the secondary antibody was taken for 30min, and incubated with primary antibody at 4°C overnight in a humidified chamber. After the following incubation with secondary antibody, DAB substrate reagent was added to the section, then observed and photographed under microscope. Primary and secondary antibodies used in this assay were listed in Supplementary Table 6.

Immunofluorescence

In this assay, HCC cells were seeded on 35mm glass bottom dish for the following corresponding treatment. After fixed in 4% paraformaldehyde and permeabilized with 0.5% Triton, 1.5% bovine serum albumin was used to block non-specific antigen for 30 min. Then, the cells were incubated with HIF-1a antibody (1:50) which diluted in block solution at 4°C overnight. Following the incubation with

Alexa Fluor® 594-conjugated secondary antibody (1:100) at room temperature for 60 min, counterstaining with DAPI was performed. Then, the samples were sealed with antifade Mounting Medium (Beyotime, China), and photographed using confocal laser scanning microscope (Olympus-FV1200, Olympus, Japan).

Luciferase reporter assay

The 3'UTR region of ETS1 mRNA and their mutant segments were cloned to construct the Luciferase reporter plasmids. Cells were seeded into 24-well plates. After cell confluency reach over 80%, these Luciferase reporter plasmids and miR-940 mimic/NC were co-transfected into the cells with Lipofectamine 3000 for 24 h. Luciferase activity was detected by luminometer according to the protocol of Dual-Luciferase® Reporter（DLR™）Assay Kit (Promega, USA).

Supplementary figure 1 PBLD Inhibits Angiogenesis Induced by Hypoxia

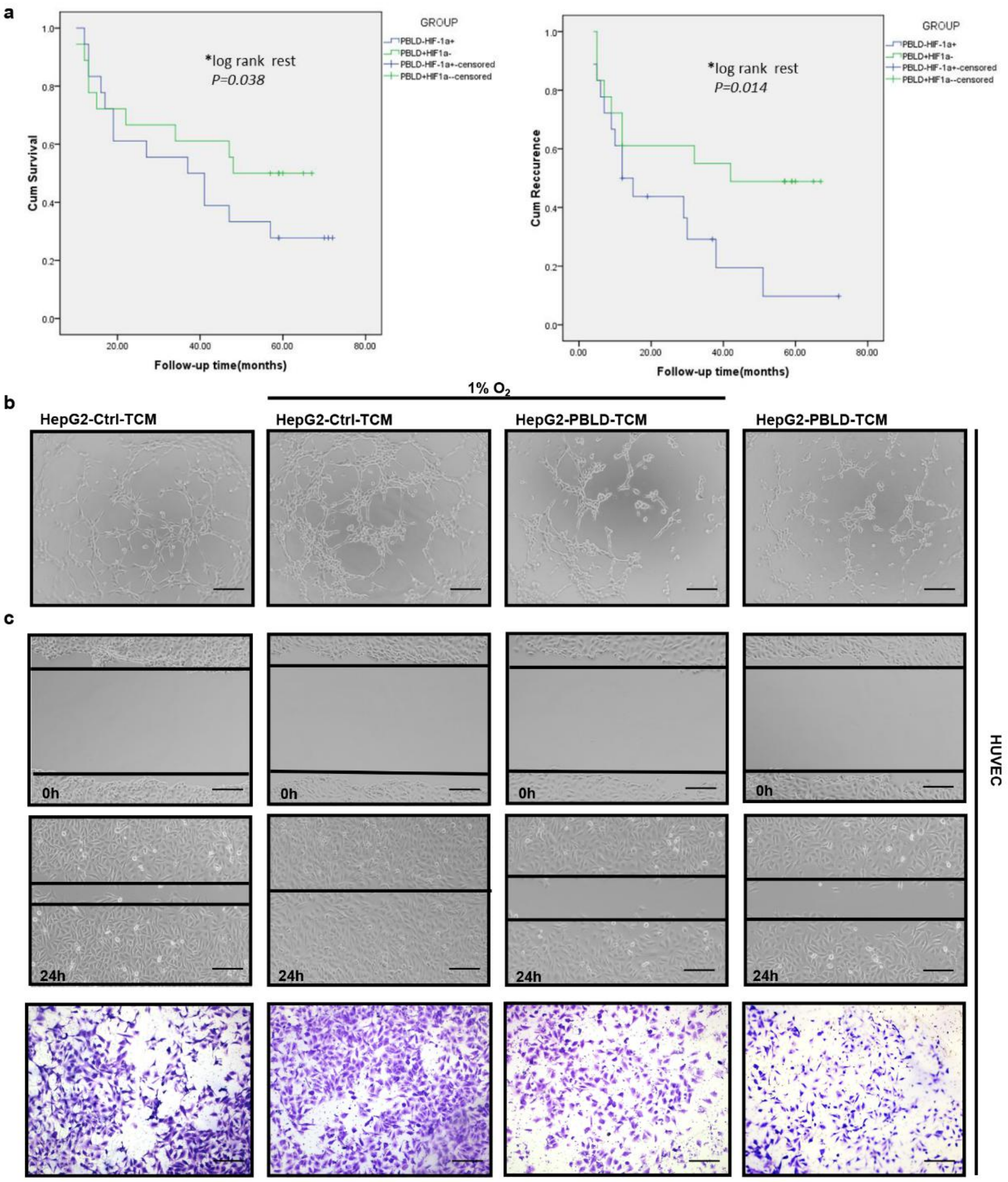

(a) Recurrence and survival analysis for 90 HCC patients. Higher expression of PBLD together with lower HIF-1a expression have a better prognosis and less recurrent. (b-c) Representative images of Transwell, wound-healing and tube-formation assay for the HUVECs which cultured in TCM from PBLD-upregulated and their control HCC cells under normal or hypoxic (1% O2) conditions. Scale bars, 200μm.

Supplementary figure 2 PBLD Suppresses HIF1-a/VEGF Axis via ERK/MAPK Signaling Pathway

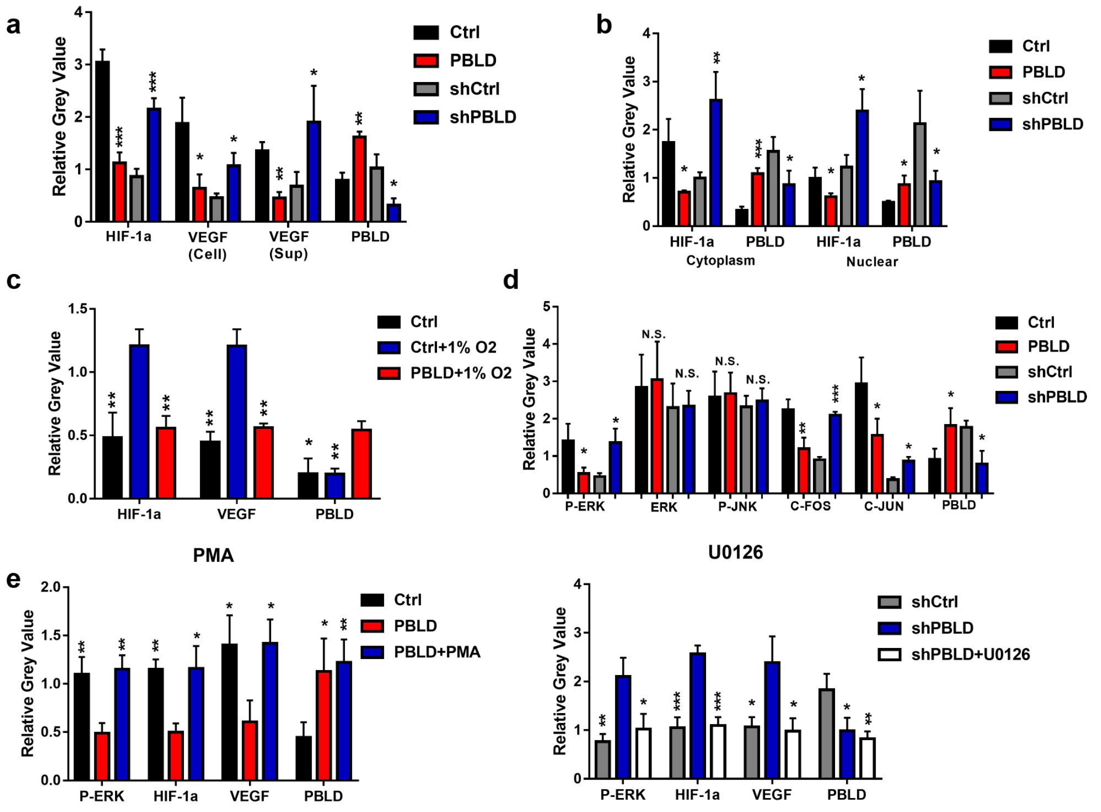

(a) The quantification of replicate western blot of VEGF and HIF-1a in HepG2 and BEL-7402 cells and the VEGF expression in the culture supernatant. (b) The quantification of replicate western blot of nuclear or cytoplasmic HIF-1a HepG2 and BEL-7402 cells. (c) The quantification of replicate western blot of VEGF and HIF-1a after PBLD upregulation in HepG2 cells under normal or hypoxic (1% O2) conditions. (d) The quantification of replicate western blot of genes in ERK/MAPK signaling pathway after PBLD upregulation or downregulation. (e) The quantification of restoration experiments results shows the impacts of ERK dephosphorylation on PBLD-mediated inhibition of HIF-1a and VEGF expression, using PMA (100nM) and U0126 (10μM).

Supplementary figure 3 PBLD Decreases ERK1/2 Phosphorylation by Protecting DUSP6 from Ubiquitin-proteasome Degradation

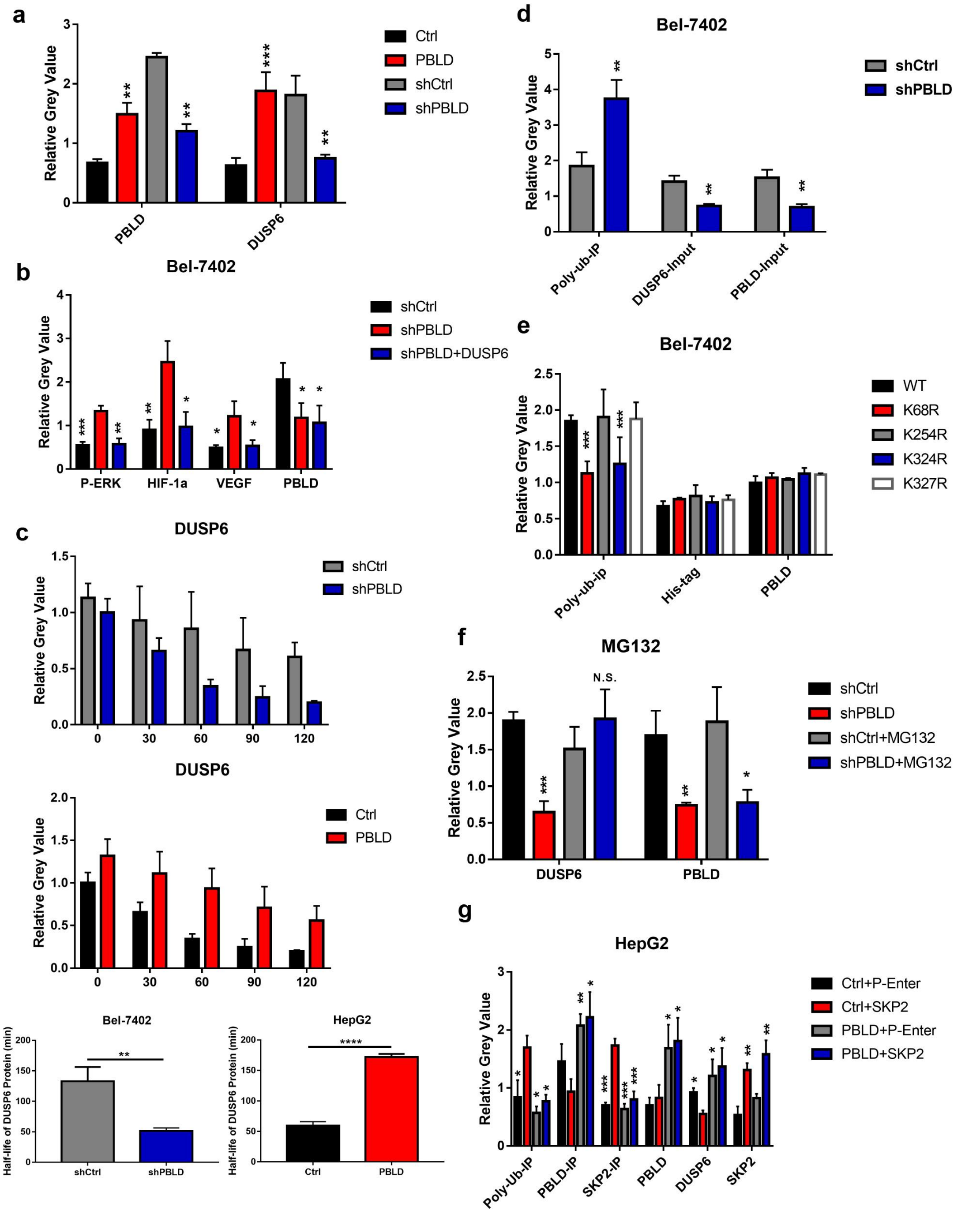

(a) The quantification of replicate western blot of DUSP6 and PBLD in HepG2 and BEL-7402 cells. (b) The quantification of replicate western blot of P-ERK, HIF-1a, VEGF, DUSP6, and PBLD in PBLD-downregulated and control group of BEL-7402 cells after transfection with DUSP6 plasmids. (c)

The quantification of replicate western blot of DUSP6 in PBLD-upregulated and PBLD-downregulated cells, which were treated with 20μg/mL CHX for the indicated periods of time. (d) The quantification of replicate western blot of co-IP assay using DUSP6 antibody in PBLD-downregulated and control HCC cells. (e) The quantification of replicate western blot of co-IP assay in PBLD-downregulated HCC cells transfected with vectors expressing the His-tagged DUSP6-WT or DUSP6-mutants (K68R, K254R, K324R, and K327R). (f) The quantification of replicate western blot of DUSP6 in PBLD-downregulated and control groups of BEL-7402 cells treated with MG132 (10μM). (g) Co-immunoprecipitation assay using DUSP6 antibody to show effects of PBLD on the combination between SKP2 and DUSP6. The quantification of replicate western blot was shown.

Supplementary figure 4 PBLD-mediated Regulation of Angiogenesis through Exosomes

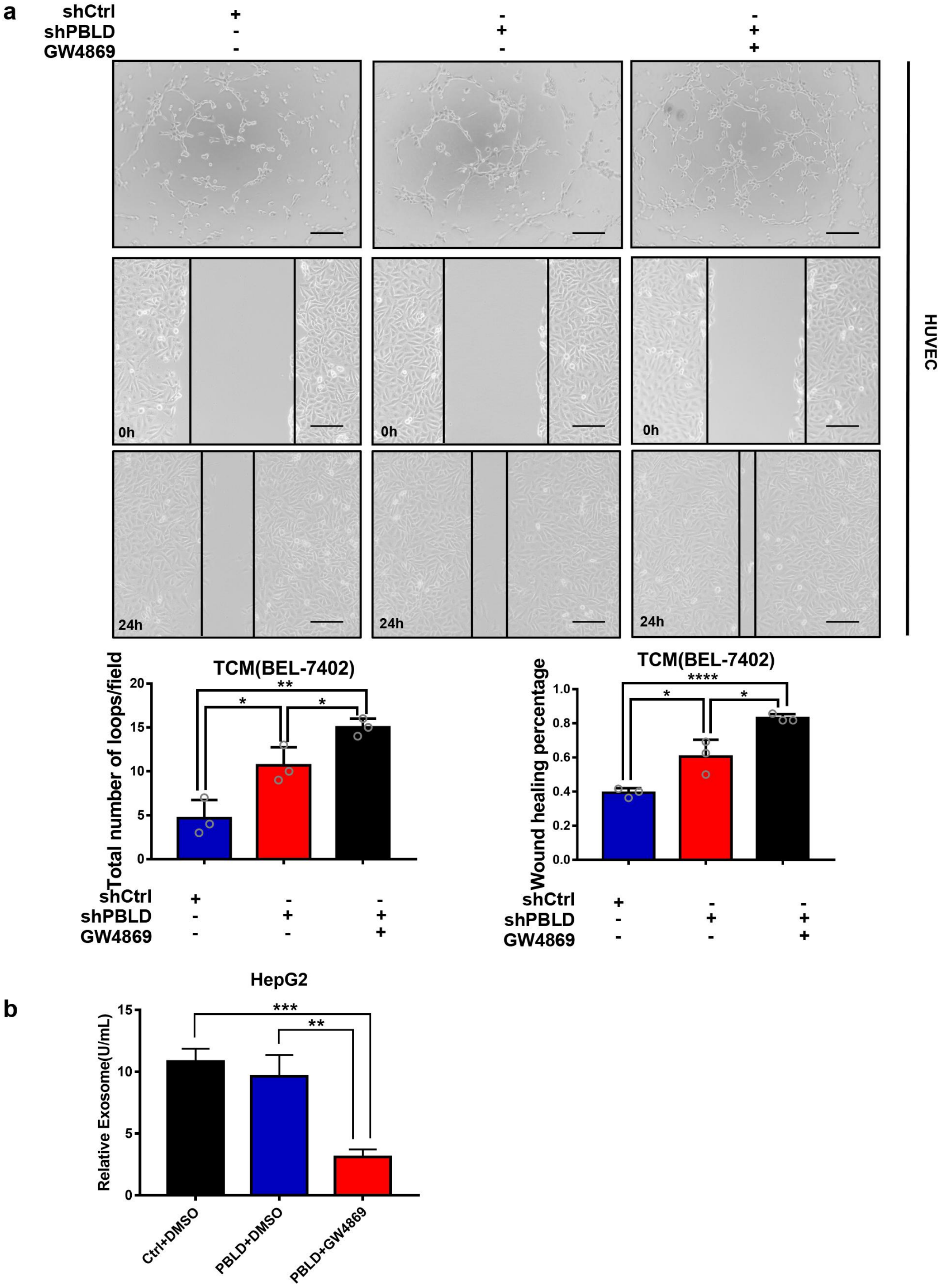

(a) Migration and tube-formation abilities of HUVECs cultured in TCM of Bel7402-shPBLD and

Bel7402-shCtrl cells pretreated with DMSO or GW4689 were detected by wound-healing and

tube-formation assays. Scale bars, 200µm. (b) The quantified results of exosomes released in different conditions by AchE assay.

Supplementary figure 5 PBLD Regulates MiR-940 Expression by Interacting with TCF4

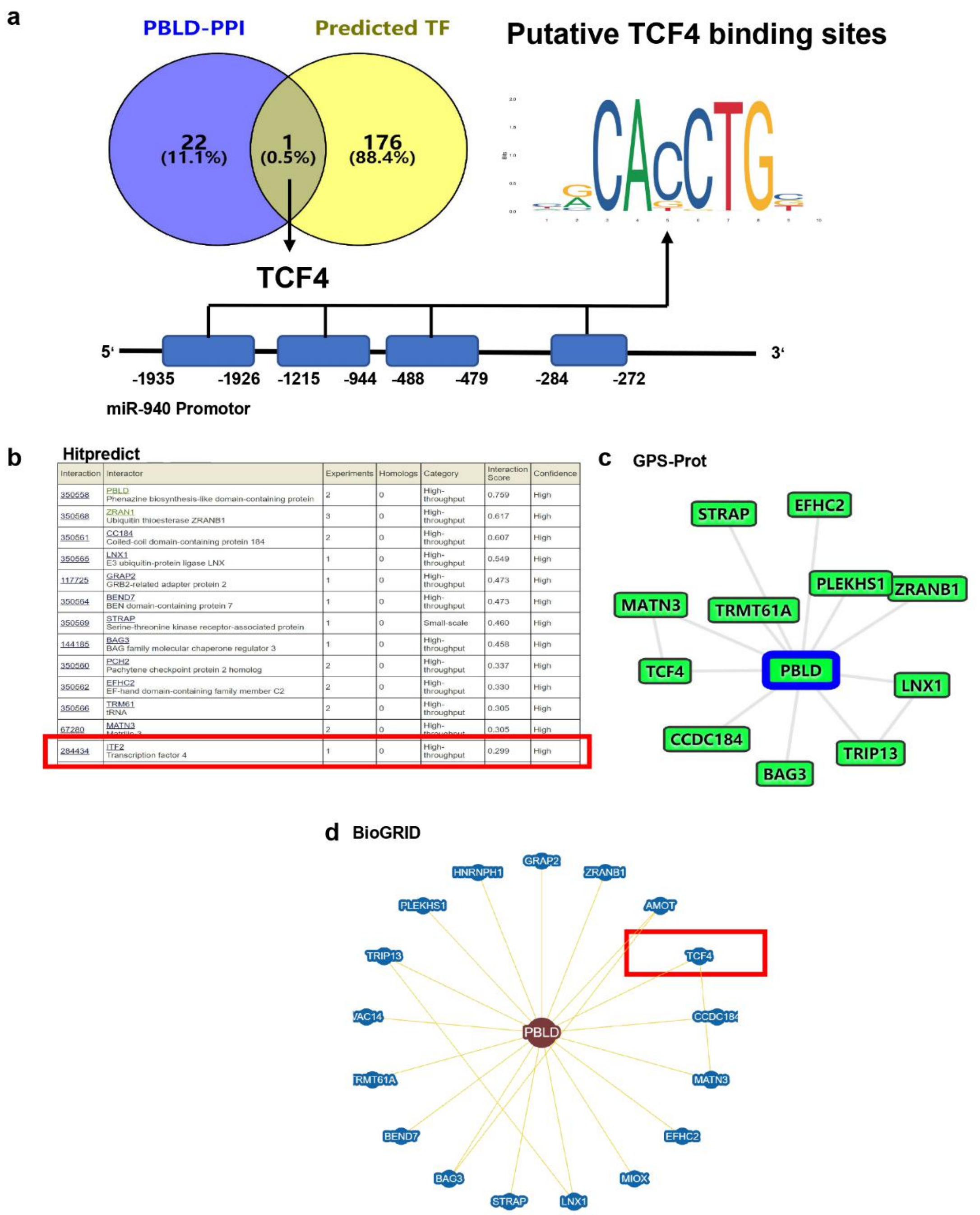

(a) The two-way Venn diagram indicates the genes that are overlapped in predicted miR-940 transcription factors (using 2 publicly available bioinformatics algorithms: TransmiR and PROMO) and proteins interacted with PBLD (using 3 online PPI websites: Hitpredict, BIOGRED and GPS-Prot). The potential TCF4-binding sites found along full-length miR-940 promoter were predicted by

bioinformatics analysis website (JASPAR). (b) Proteins predicted to interact with PBLD by Hitpredict website (http://www.hitpredict.org/). (c) Proteins predicted to interact with PBLD by GPS-Prot website (http://gpsprot.org/). (d) Proteins predicted to interact with PBLD by BIOGRID website (https://thebiogrid.org/)

Supplementary figure 6 MiR-940 Suppresses VEGF/VEGFR2 Signaling Pathway

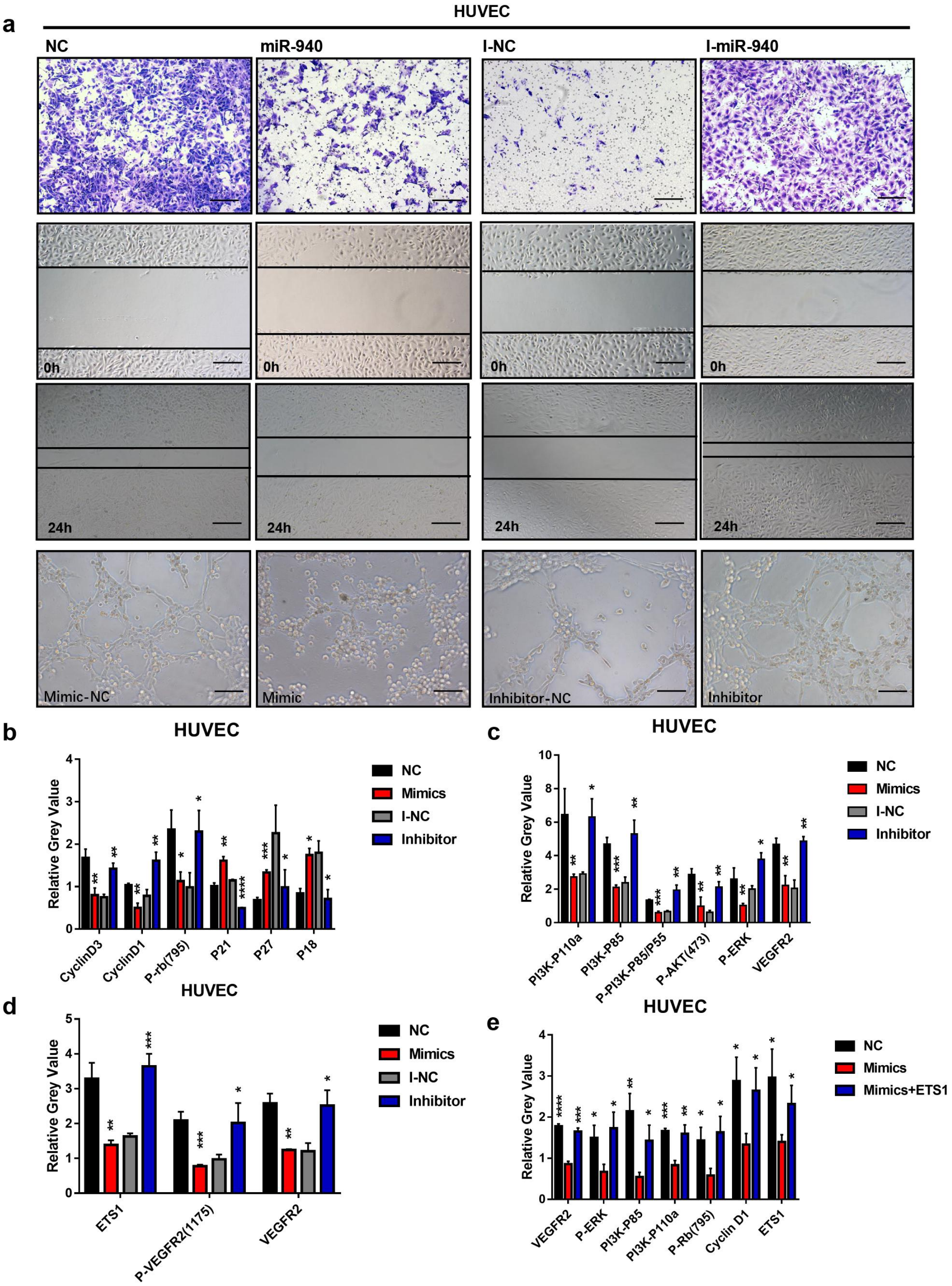

(a) The migration and tube-formation ability of HUVECs transfected with miR-940 mimics and inhibitors was detected by Transwell, wound-healing and tube formation assays. Scale bars for Transwell, wound-healing assay, 200μm; Scale bars for tube-formation assay, 100μm. (b) The

quantification of replicate western blot of cell cycle-related proteins in HUVECs transfected with miR-940 mimics and inhibitors. (c) The quantification of replicate western blot of VEGFR2 and genes downstream of VEGF/VEGFR2 in HUVECs. (d) The quantification of replicate western blot of ETS1, P-VEGFR2(1175) and VEGFR2 in HUVECs. (e) The quantification of replicate western blot of ETS1, VEGFR2 and the signaling pathways downstream of VEGF/VEGFR2 in HUVECs co-transfected with miR-940 mimics and ETS1 plasmids.

Supplementary figure 7 MiR-940 Inhibits Angiogenesis Through Regulation of VEGFR2

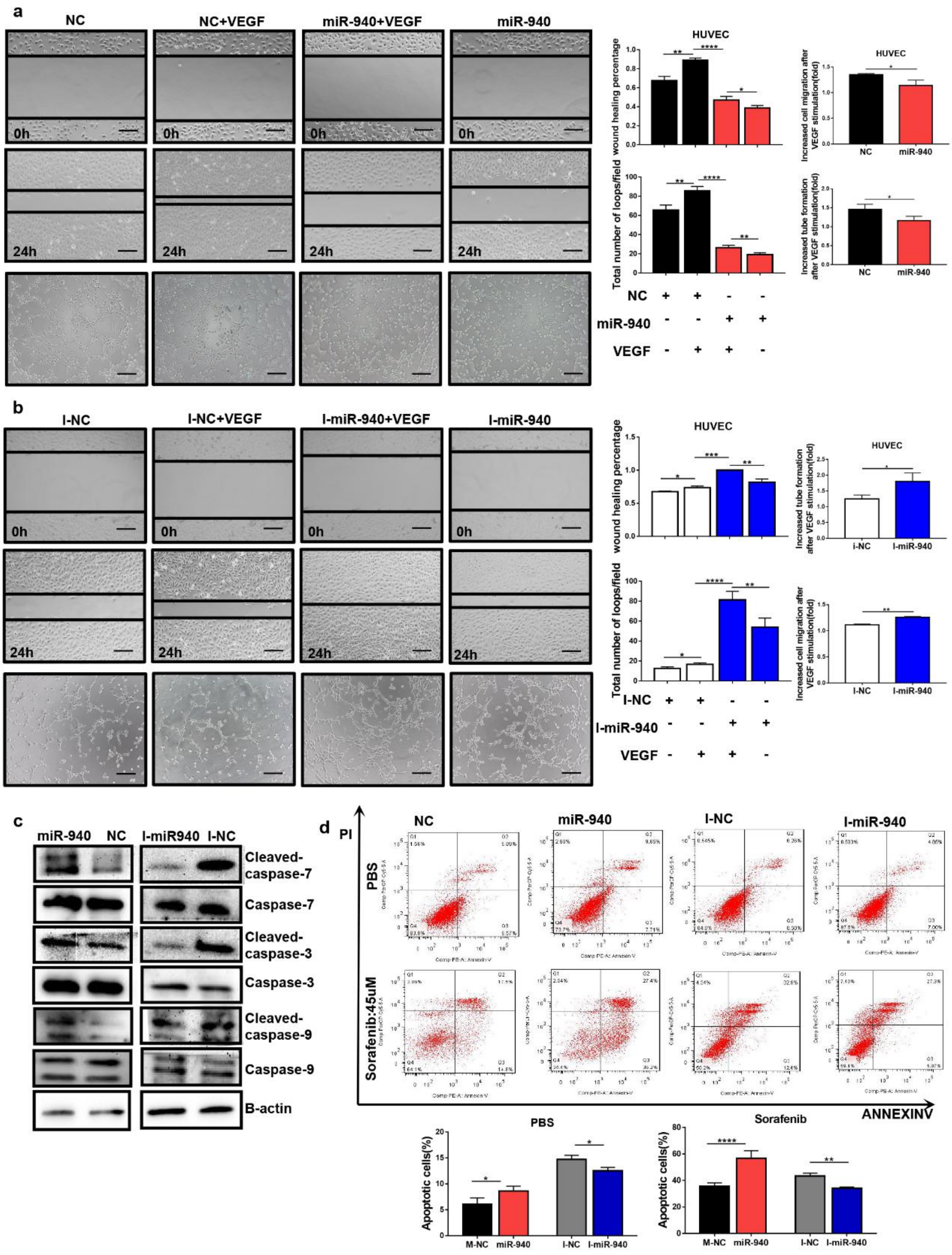

(a-b) miR-940 inhibits VEGF-induced angiogenesis. Tube-formation and migration abilities of miR-940 upregulated and downregulated HUVECs with VEGFA treatments were detected by tube-formation and wound-healing assays. Scale bars, 200μm. (c) Western blot results of

miR-940-induced activation of apoptosis signaling in HUVECs. (d) Apoptosis analysis of miR-940 upregulated and downregulated HUVECs after sorafenib treatment.

Supplementary figure 8 PBLD Inhibits Angiogenesis via Exosome/miR-940/ETS1 Axis

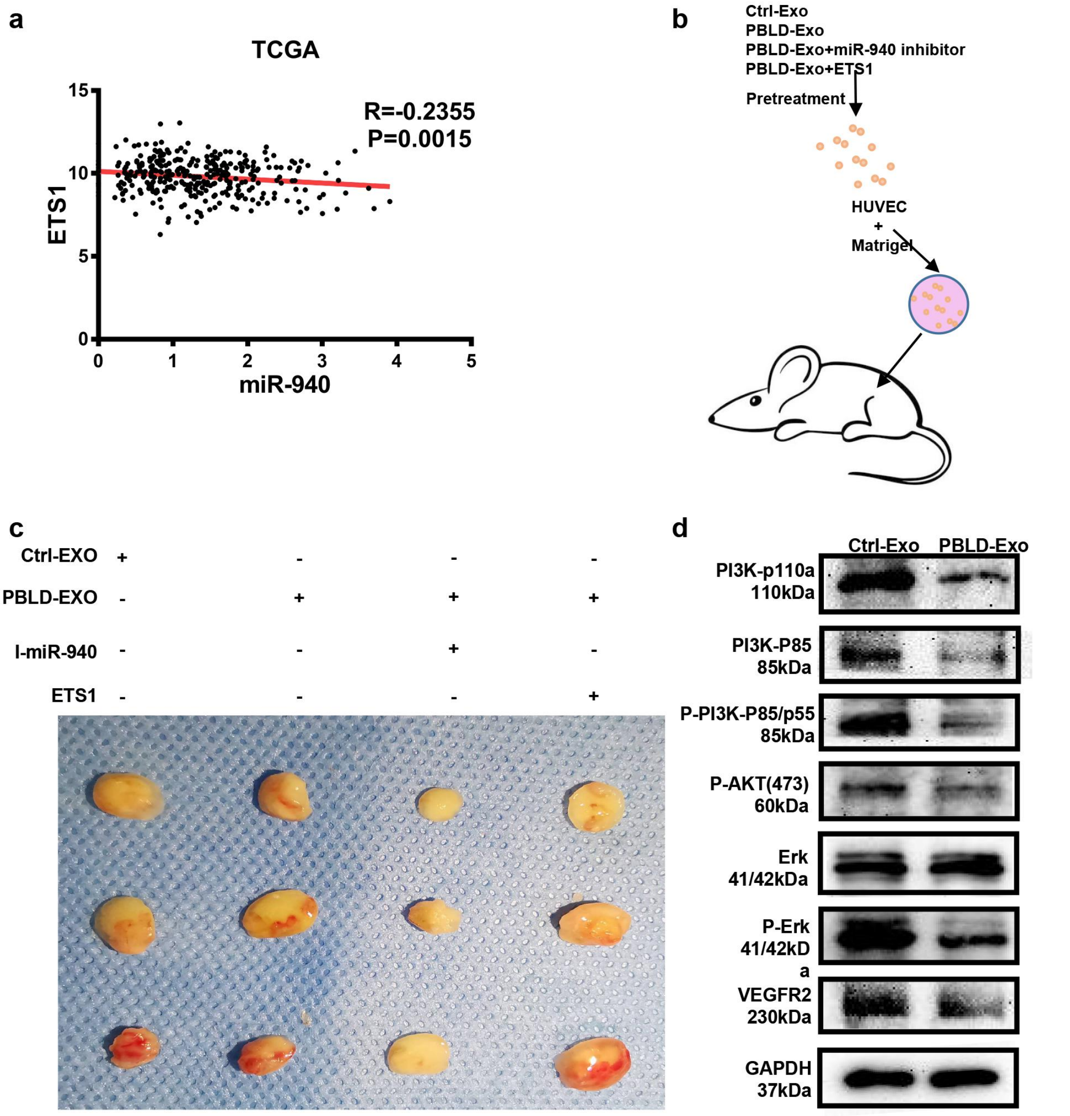

(a) Spearman's correlation analysis shows a negative correlation between ETS1 and miR-940 levels using the TCGA database (P =0.0015, r=-0.2355, n=366). (b) Schematic of the procedure for HUVECs pretreating and in vivo Matrigel plug assay. (c) The gross morphology of Matrigel plug in 4 above-mentioned groups. (d) Western blot analysis of proteins involved in angiogenesis in HUVECs after treated with exosome extracted from PBLD upregulated and control HCC cells.

Supplementary table 1 PBLD IHC score of HCC tissues and their matched normal tissues

Tissue Numbers	HCC			Normal		
	Staining intensity	positive stained area percentage	Total score	Staining intensity	positive stained area percentage	Total score
1	1	4	4	2	4	8
2	1	1	1	3	4	12
3	1	4	4	2	4	8
4	0	0	0	2	3	6
5	1	4	4	3	4	12
6	0	0	0	2	3	6
7	2	4	8	2	4	8
8	1	2	2	2	4	8
9	2	3	6	2	3	6
10	0	0	0	2	4	8
11	1	2	2	3	4	12
12	0	0	0	2	4	8
13	1	3	3	2	2	4
14	0	0	0	2	4	8
15	1	3	3	2	4	8
16	2	2	4	2	1	2
17	0	0	0	2	4	8
18	1	1	1	2	4	8
19	1	1	1	3	4	12
20	1	3	3	2	4	8
21	1	2	2	1	4	4
22	0	0	0	3	4	12
23	1	4	4	2	3	6
24	1	4	4	2	4	8
25	2	1	2	2	4	8

26	1	2	2	2	4	8
27	1	1	1	1	3	3
28	0	0	0	2	4	8
29	1	4	4	2	4	8
30	1	3	3	2	4	8
31	1	1	1	2	4	8
32	1	3	3	2	4	8
33	0	0	0	2	3	6
34	1	4	4	2	4	8
35	1	1	1	2	4	8
36	1	4	4	2	4	8
37	0	0	0	2	4	8
38	1	4	4	3	4	12
39	0	0	0	3	4	12
40	2	4	8	3	3	9
41	0	0	0	2	4	8
42	2	4	8	2	4	8
43	2	1	2	2	3	6
44	0	0	0	1	4	4
45	1	3	3	2	4	8
46	2	3	6	3	4	12
47	1	3	3	3	3	9
48	1	4	4	2	4	8
49	1	4	4	2	4	8
50	0	0	0	3	4	12
51	2	1	2	2	4	8
52	1	2	2	2	4	8
53	0	0	0	2	4	8
54	2	3	6	2	4	8
55	2	1	2	2	3	6

56	3	4	12	2	4	8
57	1	2	2	3	3	9
58	1	2	2	1	2	2
59	2	4	8	3	3	9
60	1	1	1	2	4	8
61	2	4	8	3	4	12
62	1	1	1	2	1	2
63	0	0	0	2	4	8
64	1	2	2	2	4	8
65	1	1	1	3	4	12
66	1	3	3	2	4	8
67	1	4	4	2	4	8
68	2	1	2	2	4	8
69	1	2	2	2	4	8
70	2	4	8	2	4	8
71	1	4	4	2	4	8
72	2	3	6	2	4	8
73	2	1	2	3	4	12
74	2	2	4	3	4	12
75	1	4	4	3	3	9
76	0	0	0	2	4	8
77	1	3	3	2	4	8
78	0	0	0	3	4	12
79	2	2	4	3	3	9
80	2	4	8	2	3	6
81	1	2	2	1	4	4
82	2	4	8	2	2	4
83	1	3	3	3	2	6
84	0	0	0	2	4	8
85	1	2	2	2	3	6

86	2	3	6	2	4	8
87	1	2	2	2	4	8
88	0	0	0	2	4	8
89	0	0	0	2	4	8
90	0	0	0	1	1	1

Supplementary table 2 HIF-1a IHC score of HCC tissues

Tissue Numbers	HCC		
	Staining intensity	Positive stained area percentage	Total score
1	0	0	0
2	1	1	1
3	0	0	0
4	1	1	1
5	0	0	0
6	0	0	0
7	0	0	0
8	0	0	0
9	1	2	2
10	2	2	4
11	1	1	1
12	2	4	8
13	3	4	12
14	1	1	1
15	0	0	0
16	2	2	4
17	1	2	2
18	0	0	0
19	3	4	12
20	2	2	4
21	0	0	0
22	2	4	8
23	1	4	4
24	0	0	0
25	1	1	1
26	0	0	0

27	2	3	6
28	3	4	12
29	2	3	6
30	0	0	0
31	1	1	1
32	0	0	0
33	2	3	6
34	1	1	1
35	1	2	2
36	2	4	8
37	1	1	1
38	0	0	0
39	2	2	4
40	1	1	1
41	3	4	12
42	1	1	1
43	3	3	9
44	0	0	0
45	0	0	0
46	2	2	4
47	0	0	0
48	0	0	0
49	2	1	2
50	1	1	1
51	3	4	12
52	3	4	12
53	2	3	6
54	3	4	12
55	1	1	1
56	1	1	1

57	2	2	4
58	1	1	1
59	1	1	1
60	0	0	0
61	3	4	12
62	0	0	0
63	1	1	1
64	2	2	4
65	3	4	12
66	1	2	2
67	1	2	2
68	1	2	2
69	2	2	4
70	0	0	0
71	0	0	0
72	0	0	0
73	1	2	2
74	2	2	4
75	1	1	1
76	1	1	1
77	3	4	12
78	1	1	1
79	1	2	2
80	1	3	3
81	2	4	8
82	1	1	1
83	1	1	1
84	2	2	4
85	3	4	12
86	2	3	6

87	2	2	4
88	0	0	0
89	3	4	12
90	1	1	1

Supplementary table 3 Predicted E3 ligases for DUSP6

E3 ligases	Enriched domain pair	E3 recognizing motif	Network loops	Ortholog interaction	Enriched GO pair	Confidence Level	Score
SKP2	√	√	√		√	HIGH	0.766
NEDD4		√	√		√	MIDDLE	0.743
MARCH1		√			√	MIDDLE	0.738
RNF216		√			√	MIDDLE	0.714
CBL		√	√		√	MIDDLE	0.696
STUB1			√		√	MIDDLE	0.691
SYVN1		√	√		√	MIDDLE	0.687
BTRC	√	√	√		√	MIDDLE	0.686
FBXW7	√		√		√	MIDDLE	0.681
FBXO11	√		√		√	MIDDLE	0.681
FBXW5	√		√			MIDDLE	0.679
FBXL6	√		√		√	MIDDLE	0.671
FBXL12	√		√		√	MIDDLE	0.670
FBXW11	√		√		√	MIDDLE	0.670

Another 50 predicted E3 ligases for DUSP6 in low confidence level are not listed in this table.

Supplementary table 4 List of upregulated microRNAs in exosome from HepG2 cells by comparing the PBLD-overexpression group and the control group

Systematic Name	miRbase accession No.	p-value	Fold change	P1-NS	P2-NS	P3-NS	C1-NS	C2-NC	C3-NS
hsa-miR-7110-5p	MIMAT0028117	0.014540995	6.400896963	9.012146085	9.016693943	9.628219116	6.299211945	5.209495657	7.002120329
hsa-miR-6740-5p	MIMAT0027381	0.04948409	4.77481887	6.710428139	6.002006022	7.178543289	4.12904744	3.765386916	4.409649212
hsa-miR-574-5p	MIMAT0004795	0.026788899	4.518885332	4.196880052	5.490156894	6.384834761	3.845598167	3.719113928	3.353573323
hsa-miR-4800-5p	MIMAT0019978	0.034853737	4.483234513	6.820238749	5.437700094	5.998353505	3.634107712	3.492345034	4.027975387
hsa-miR-4644	MIMAT0019704	0.000270109	4.208321102	4.702003025	4.800159202	4.899536012	2.451268995	2.484977006	2.906214322
hsa-miR-671-5p	MIMAT0003880	0.026810381	4.201149071	7.115108989	6.908527904	5.757020966	4.340280119	3.744168547	4.792625692
hsa-miR-940	MIMAT0004983	0.010164252	4.032574844	5.664347218	7.097540058	5.908189267	4.314477497	4.035147867	4.24493188
hsa-miR-4665-3p	MIMAT0019740	0.01651228	3.693991729	5.601783694	6.861540502	5.188414161	4.181119225	3.988817175	4.030598565
hsa-	MIMAT0005589	0.04900356	3.635432042	4.3985	6.7067	4.8407	3.6983	3.6539	3.9008

miR-1234-3p				0384	4505	80247	93573	95635	97262
hsa-miR-3162-3p	MIMAT0019213	0.026566953	3.473235497	4.227150108	5.968414141	4.290535312	3.343891966	3.117900695	3.47531316
hsa-miR-6797-3p	MIMAT0027495	0.040713796	3.437611022	4.108082508	6.210242707	4.463200984	3.39684616	3.274557201	3.629698787
hsa-miR-6076	MIMAT0023701	0.047398808	3.36328579	6.196124846	6.363381285	5.510014006	4.003586272	3.455148273	4.442690177
hsa-miR-1228-3p	MIMAT0005583	0.037734875	3.216465986	4.226797965	6.237262149	4.671223805	3.56910548	3.399321996	3.750170082
hsa-miR-6069	MIMAT0023694	0.047007815	3.13172337	3.983665048	6.05400927	4.355978958	3.399859333	3.189521528	3.569096539
hsa-miR-1238-3p	MIMAT0005593	0.02592209	2.657537654	3.847668022	5.322560153	4.177727042	3.178456488	3.140913686	3.241900363
hsa-miR-1304-3p	MIMAT0022720	0.041390331	2.397033526	3.712244228	5.3250253	4.071940024	3.202502448	3.025911905	3.405084991
hsa-miR-6508-5p	MIMAT0025472	0.026749888	2.36567644	3.678455779	5.042935586	3.970042847	3.152586312	2.975282293	3.199623032
hsa-miR-6737-3p	MIMAT0027376	0.014963978	2.33442209	3.859876464	4.778323468	3.717146529	2.994039834	2.933733968	3.112421757
hsa-	MIMAT0027603	0.030852961	2.313337565	3.4542	4.8751	3.9042	2.9667	2.8846	3.0623

miR-6851-3p				47063	81532	77775	50811	75237	25447
hsa-miR-4725-5p	MIMAT0019843	0.032674719	2.288039416	3.444712309	4.932903672	3.89338303	3.006773761	2.823726368	3.244445963
hsa-miR-4649-3p	MIMAT0019712	0.015892954	2.275007575	3.585054299	4.664630572	3.66848624	2.812284765	2.744668115	2.981917122
hsa-miR-6515-3p	MIMAT0025487	0.011709828	2.172691729	3.46466976	4.529202592	3.833742671	2.848081175	2.78464867	3.121112559
hsa-miR-6813-3p	MIMAT0027527	0.008480575	2.08769361	3.443176092	4.23703406	3.522274339	2.686248793	2.63148541	2.776258047
hsa-miR-4313	MIMAT0016865	0.034712534	2.082209941	3.471018346	4.791645304	3.856075561	3.128273282	2.927483511	3.160821597
hsa-miR-6819-3p	MIMAT0027539	0.002478422	2.056400202	3.522064957	4.169939841	4.076327981	2.982985817	2.767816022	2.969115133
hsa-miR-6880-3p	MIMAT0027661	0.024060448	2.008758178	3.540703614	4.324355177	3.218735569	2.706467393	2.682884933	2.834798844
hsa-miR-6889-3p	MIMAT0027679	0.028690031	1.921461367	3.566729535	4.41058021	3.278088121	2.872118076	2.706964358	3.009144691
hsa-miR-4769-3p	MIMAT0019923	0.026812919	1.76894452	3.008048918	3.815598819	3.041950194	2.625232328	2.513284262	2.568860917
hsa-	MIMAT0027641	0.003854853	1.442573578	2.7825	2.9680	2.6056	2.4012	2.1654	2.2833

miR-6870-3p	81988	58617	66189	53356	13162	57354

This table only shows the upregulated microRNAs that have a foldchange greater than 1.

Supplementary table 5 The sequences of PCR primers and siRNAs

Genes		Sequences
ChIP-qRT-PCR		
-217~-365	F	CGTTGGATTTGGGTTTGA
	R	GGCATGGGCTCTAGGTTT
-479~-488	F	GCAGGCATAGGCGGAATT
	R	GCCCACCAGGTGTCAGT
-944~-1215	F	ATGGAGGGCATTAGGCAGTG
	R	AGGATGGAGGCAGGGAGGA
-1926~-1935	F	GGAGTGGGTATCATCAGGG
	R	GAATCACCAAGGCTCATCTAG
qRT-PCR		
PBLD	F	TCCTGCTTTGGACTGAGATGG
	R	CGTGAGCGTGCTATTCATGTTTT
CD31	F	AACAGTGTTGACATGAAGAGCC
	R	TGTAAAACAGCACGTCATCCTT
ETS1	F	GATAGTTGTGATCGCCTCACC
	R	GTCCTCTGAGTCGAAGCTGTC
VEGFR2	F	TTTGGCAAATACAACCCTTCAGA
	R	GCAGAAGATACTGTCACCACC
DUSP6	F	GAACTGTGGTGTCTTGGTACATT

	R	GTTCATCGACAGATTGAGCTTCT
TCF4	F	TGCAAAGCCGAATTGAAGATCG
	R	AGAAGGTCCAATGATTCCATGC
MiR-940	F	AAGGCAGGGCCCCCGCTCCCC
	R	Provided by Mir-XTM miRNA First-Strand Synthesis Kit (Takara, USA) -- (miRNA-specific primer)
U6	F	Provided by Mir-XTM miRNA First-Strand Synthesis Kit (Takara, USA) -- (U6 Forward Primer)
	R	Provided by Mir-XTM miRNA First-Strand Synthesis Kit (Takara, USA) -- (U6 Reverse Primer)
GAPDH	F	ACAACTTTGGTATCGTGGAAGG
	R	GCCATCACGCCACAGTTTC
Mimics and inhibitors for miR-940		
Mimics	5' to 3'	AAGGCAGGGCCCCCGCUCCCC
		GGAGCGGGGGCCCUGCCUUUU
NC	5' to 3'	UUCUCCGAACGUGUCACGUTT
		ACGUGACACGUUCGGAGAATT
Inhibitors	5' to 3'	GGGGAGCGGGGGCCCUGCCUU
Inhibitors NC	5' to 3'	CAGUACUUUUGUGUAGUACAA

Supplementary table 6 List of primary and secondary antibodies used in the study

Antibody	Applications	Company
PBLD	WB, IHC	Abcam (ab235947)
PBLD	WB, IP	Santa Cruz (sc-101502)
β-Actin	WB	Proteintech (66009-1-Ig)
α-tubulin	WB	Proteintech (66031-1-Ig)
GAPDH	WB	Proteintech (60004-1-Ig)
CD31	IHC	Cell Signaling Technology (3528)
HIF-1a	WB, IF	Novus Biologicals (NB100-105)
HIF-1a	WB, IHC	Cell Signaling Technology (3716)
VEGF	WB	Santa Cruz (sc-7269)
VEGF	Neutralization	Thermo Fisher (26503)
DUSP6	WB, IP	Abcam (ab76310)
Lamin B1	WB	Proteintech (12987-1-AP)
ERK	WB	Cell Signaling Technology (4695)
P-ERK	WB	Cell Signaling Technology (4370)
C-FOS	WB	Abcam (ab222699)
C-JUN	WB	Abcam (ab32137)

SKP2	WB	Proteintech (15010-1-AP)
His-tag	WB, IP	Proteintech (66005-1-Ig)
Ubiquitin	WB	Proteintech (10201-2-AP)
CD63	WB	SBI (EXOAB-CD63A-1)
CD9	WB	SBI (EXOAB-CD9A-1)
HSP70	WB	SBI (EXOAB-Hsp70A-1)
VEGFR2	WB	Cell Signaling Technology (9698)
PI3K p85a	WB	Cell Signaling Technology (4257)
PI3K p110a	WB	Cell Signaling Technology (4249)
P-PI3K-p85/p55	WB	Cell Signaling Technology (4228)
p-AKT (473)	WB	Cell Signaling Technology (4060)
Cyclin D1	WB	Cell Signaling Technology (2978)
Cyclin D3	WB	Cell Signaling Technology (2936)
P21	WB	Cell Signaling Technology (2947)
P18	WB	Cell Signaling Technology

		(2890)
P27	WB	Cell Signaling Technology (3686)
P-Rb (795)	WB	Cell Signaling Technology (4539)
Caspase 3	WB	Cell Signaling Technology (14220)
Cleaved caspase 3	WB	Cell Signaling Technology (9664)
Caspase 7	WB	Cell Signaling Technology (12827)
Cleaved caspase 7	WB	Cell Signaling Technology (8438)
Caspase 9	WB	Cell Signaling Technology (9508)
Cleaved caspase 9	WB	Cell Signaling Technology (52873)
TCF4	WB, IP, ChIP	Abcam (ab217668)
ETS1	WB	Abcam (ab220361)
Alexa Fluor® 594-conjugated goat anti-mouse IgG (H + L)	IF	Zhongshan Biotech (ZF-0513)
Anti-mouse IgG, HRP-linked Antibody	WB	Cell Signaling Technology (7076)

Anti-rabbit IgG, HRP-linked Antibody	WB	Cell Signaling Technology (7074)
Mouse Anti-rabbit IgG (Conformation Specific) (L27A9) mAb (HRP Conjugate)	WB	Cell Signaling Technology (5127)

Abbreviations: ChIP, chromatin immunoprecipitation; IF, immunofluorescence; IHC, immunohistochemistry; WB, western blot.

Supplementary table 7 Clinicopathologic features of patients in Nanfang cohort

ID	Differentiation	Age	Sex	AFP	HBsAg	Cirrhosis	Tumor size	CD31	PBLD
ZA3406887	Moderate	43	Male	4716.1ug/L	Positive	Nodular cirrhosis	3.8	0.0279722	0.04615
ZA3334160	Moderate	54	Male	7310.6ug/L	Positive	Cirrhosis	8	0.064718	0.027436
ZA3776526	Moderate	46	Male	2.8ug/L	Positive	Nodular cirrhosis	3	0.022689	0.082861
33004202	Poor	67	Male	61.9ug/L	Positive	Nodular cirrhosis	3	0.1610094	0.001223
ZA2692715	Well to moderate	83	Male	14.7ug/L	Negative	No	7	0.0456166	0.056142
ZA3380693	Poor	62	Male	100.3ug/L	Positive	Nodular cirrhosis	1.7	0.1142728	0.001867
33027560	Well	49	Male	10.2ng/ml	Positive	Hepatic steatosis	1.6	0.0365421	0.039467
33041038	Well	57	Male	6.7ug/L	Positive	Early cirrhosis	15	0.0451356	0.28635
YB0119170	Moderate to poor	40	Male	16994.7ug/L	Negative	Early cirrhosis	6	0.027877	0.062973
33041862	Moderate	36	Male	34.1ug/L	Positive	No	11	0.0085282	0.013094
ZA3855979	Moderate to poor	70	Male	2774.5ug/L	Positive	Nodular cirrhosis	3	0.0220208	0.009606
yb0141946	Well	39	Male	70632.1ug/L	Positive	Nodular cirrhosis	8	0.0209839	0.032804
33047318	Moderate to poor	47	Male	>1000ng/ml	Positive	No	22	0.0455969	0.032584
za224624	Well	44	Male	98.8ug/ml	Positive	Nodular cirrhosis	5	0.059994	0.007366
yb0142833	Well	39	Male	168.2ug/L	Positive	Nodular cirrhosis	2	0.08599	0.00144
YB018129	Well	63	Male	unknown	Negative	Early cirrhosis	6	0.0063093	0.022177
YB018132	Well	45	Male	9.0ug/L	Positive	Nodular cirrhosis	5	0.0945321	0.03642
YB0181505	Well	40	Male	9.9ug/L	Positive	No	10	0.041378	0.004218
YB0175609	Well	14	Female	80408.9ug/L	Positive	Early cirrhosis	10	0.0018808	0.033916
ZA2724290	Well	64	Male	11.7ug/L	Positive	Early cirrhosis	3	0.0305232	0.00284

ZA2247644	Poor	45	Male	3340ng/ml	Positive	Early cirrhosis	3	0.0181841	0.031839
ZA2524887	Poor	44	Male	>100000ug/L	Positive	Nodular cirrhosis	8	0.0369202	0.015146
ZA2603761	Poor	40	Male	>100000ug/L	Positive	Cirrhosis	11.5	0.012582	0.044246
QA0017715	Well	66	Female	1168.7ug/L	Negative	Nodular cirrhosis	3	0.0379125	0.002982
ZA2798010	Well	57	Male	21.3ng/L	Unkown	Early cirrhosis	9	0.027112	0.05852
ZA2824667	Poor	33	Male	834ug/L	Positive	Cirrhosis	17	0.0112434	0.003185
ZA3058098	Well	64	Male	10.5ug/L	Positive	Unkown	6.5	0.1456232	0.000105
ZA3010958	Well	46	Male	7.3ug/L	Positive	Nodular cirrhosis	5	0.1391731	0.000836
QA0018609	Well	36	Female	unknown	Positive	No	6	0.0178591	0.003521
33483326	Well	25	Male	51223.3ug/L	Positive	No	7	0.1375593	0.002653
33482121	Moderate	36	Male	48100.8ug/L	Positive	Unkown	11	0.0598762	0.001881
33483708	Poor	66	Male	>100000ug/L	Positive	Micronodular cirrhosis	8	0.0499083	0.000862
33488175	Moderate	49	Male	5554ug/L	Positive	Early cirrhosis	9.5	0.0880831	0.000387
33490966	Moderate	58	Male	37164.8ug/L	Positive	No	10	0.01418	0.062781
33508109	Moderate	70	Male	133.7ug/L	Negative	Nodular cirrhosis	7.5	0.0316862	0.078563
QA0022927	Moderate	65	Male	3795.8ug/L	Positive	No	Unkown	0.0128686	0.103307
ZA2773383	Poor to moderate	32	Female	29504.3ug/L	Positive	Unkown	9	0.0235195	0.125471
33672765	Well	56	Male	9.3ug/L	Positive	Unkown	7.2	0.0555527	0.427821
ZA2569064	Modetate	67	Female	1140.8ug/L	Positive	Nodular cirrhosis	2	0.0188407	0.358523
ZA0041293	Well	37	Male	605.8ug/L	Positive	Nodular cirrhosis	3	0.0245183	0.211168
ZA2943931	Moderate to poor	45	Male	607.4ng/ml	Positive	Nodular cirrhosis	13.4	0.013697	0.157127
ZA27769	Moderate	64	Fema	30.4ug/L	Positiv	Nodular	3	0.00407	0.1862

638			le		e	cirrhosis		21	11
ZA2790744	Moderate	46	Female	>100000ug/L	Positive	Cirrhosis	11.2	0.0109722	0.439954
ZA2834091	Moderate	44	Female	28992.4ug/L	Positive	No	15	0.0102374	0.246132
ZA2826713	Moderate	62	Male	2426.2ug/L	Positive	Nodular cirrhosis	2.3	0.0225614	0.098132
ZA2833495	Moderate	53	Male	>100000ug/L	Positive	Cirrhosis	15	0.0344345	0.124784
ZA2835026	Moderate	32	Male	66.1ug/L	Positive	Nodular cirrhosis	8	0.042101	0.098428
ZA2864246	Moderate	33	Male	>100000ug/L	Positive	No	12	0.0030017	0.034715
ZA2973007	Moderate	56	Male	2943.4ug/L	Positive	Nodular cirrhosis	3	0.0448111	0.001603
YB0053015	Poor	44	Male	>100000ug/L	Positive	Unkown	Unkown	0.0207607	0.111881
ZA2976450	Moderate	45	Male	19674.2ug/L	Negative	Early cirrhosis	7	0.0567199	0.142103
ZA2584572	Moderate	68	Male	7889.5ug/L	Positive	Nodular cirrhosis	14.5	0.0012276	0.138583
ZA3079201	Moderate	48	Male	4.3ug/L	Positive	No	2	0.0438889	0.082196
ZA2746471	Moderate to poor	36	Male	6956ug/L	Positive	Nodular cirrhosis	12	0.00709	0.303556
ZA3051254	Well to moderate	70	Male	107.5ug/L	Positive	Nodular cirrhosis	8.5	0.0158431	0.012003
ZA3044689	Well to moderate	53	Male	2255.96ug/L	Positive	Nodular cirrhosis	3	0.0097526	0.227229
QA0018027	Moderate	30	Male	15585.6ug/L	Positive	Nodular cirrhosis	4.6	0.0179484	0.186211
ZA3093322	Moderate	50	Male	5.7ug/L	Positive	Nodular cirrhosis	2	0.0066612	0.204495
ZA3021442	Moderate	56	Male	300.44ng/ml	Positive	No	10	0.0547879	0.136314
ZA2527987	Well to moderate	39	Male	426ug/L	Negative	Nodular cirrhosis	9	0.0095519	0.142294
ZA3049282	Moderate	35	Male	236.4ug/L	Positive	Nodular cirrhosis	7	0.0203335	0.002931
ZA250569	Well	65	Male	45.3ug/L	Positive	Nodular cirrhosis	10	0.0134151	0.006449
ZA3170414	Moderate to poor	64	Male	1540.9ug/L	Positive	Early cirrhosis	4	0.0128686	0.004158
QA00182	Well	75	Male	16973.4ug	Positiv	Nodular	7	0.07432	0.0016

80				/L	e	cirrhosis		54	49
ZA3161310	Moderate	47	Male	7.7ug/L	Positive	No	12.2	0.0555527	0.002152
ZA3176301	Moderate	48	Male	107.6ug/L	Positive	Nodular cirrhosis	4.5	0.0733022	0.001953
ZA3143495	Moderate	30	Male	509.4ug/L	Positive	No	7	0.0279695	0.00205
QA0018584	Moderate	60	Male	57.3ug/L	Positive	Portal cirrhosis	6	0.0255594	0.004581
ZA3163056	Moderate	48	Male	1503.7ug/L	Positive	Nodular cirrhosis	4	0.0438889	0.005186
ZA0676437	Moderate	48	Male	3664.9ug/L	Positive	Nodular cirrhosis	2.5	0.0118415	0.004249
